# Novel mitochondrial-targeted alkyl chains act as fungal specific inhibitors of *C. neoformans*

**DOI:** 10.3389/fmicb.2024.1505308

**Published:** 2025-02-04

**Authors:** Elizabeth S. M. Edrich, Luke Young, John Spencer, Andrew McGown, Anthony L. Moore, Campbell W. Gourlay

**Affiliations:** ^1^Kent Fungal Group, School of Biosciences, University of Kent, Kent, United Kingdom; ^2^Sussex Drug Discovery Centre, School of Life Sciences, University of Sussex, Brighton, United Kingdom

**Keywords:** cryptococcus, drug, mitochondria, alkyl, antifungal

## Abstract

*Cryptococcus neoformans* is the causal agent of cryptococcal meningitis in immunocompromised patients and increasing instances of anti-fungal resistance have led to investigations into new alternative antifungal targets. For example, *C. neoformans* possesses an Alternative Oxidase enzyme (Aox) that has been implicated in stress resistance and virulence that may represent a viable antifungal target. Here we test the efficacy of mitochondrially-targeted Colletochlorin B, which has been shown to inhibit the Aox of *Candida albicans in vitro*. Two derivatives of Colletochlorin B, which we modified to improve delivery to mitochondria, were identified as putative fungal-specific inhibitors. ALTOX094 and ALTOX102 were able to inhibit Aox and cytochrome *bc*_1_*in vitro* and demonstrated strong inhibitory effects against *C. neoformans* growth and viability. Further analysis suggested that the antifungal properties of ALTOX094 and ALTOX102 were attributable to different modes of action and forms of cell death, governed largely by the alkyl chain length used to tether Colletochlorin B to the mitochondria targeting triphenylphosphine (TPP) moiety. Our findings add to the growing evidence that functionalized mitochondria targeted alkyl chains may developed further as an effective class of antifungal and are effective against *C. neoformans*.

## Introduction

*Cryptococcus neoformans* is an opportunistic fungal pathogen that affects immunocompromised individuals such as those with HIV or patients treated with immunosuppressant drugs (Rajasingham et al., 2023). It is estimated that there are 223,100 cases of AIDS related cryptococcal meningitis per year, attributing 19% of global AIDS related deaths (Rajasingham et al., 2023), with most cases surfacing in sub-Saharan Africa and countries such as Brazil and Thailand (Youbao et al., 2023; Hansakon et al., 2019; do Carmo et al., 2022). However, the varied nature of symptoms presented by patients infected with *C. neoformans* can lead to prolonged misdiagnosis, and inconsistencies in the maintenance of treatment programmes has led to increases in antifungal resistance (William et al., 2023; Bermas and Geddes-McAlister, 2023). This includes *C. neoformans* heteroresistance to Fluconazole (Stone et al., 2019) and evidence of resistance providing cross-tolerance to other drug classes through aneuploidy (Feng et al., 2023). Anti-fungal resistance within *C. neoformans* populations has resulted in the search for new alternative anti-fungal targets, including mitochondria, which are thought to contribute to cryptococcal pathogenesis (Black et al., 2021; Edrich et al., 2024; Moore et al., 2013).

The fungal respiratory chain and its potential as an antifungal target has recently been reviewed (Edrich et al., 2024; Szibor et al., 2022). Many of the Electron Transport Chain (ETC) components are well-conserved among both fungi and humans, however, fungal specific components that may represent viable drug targets do exist. For example, many fungi possess an Alternative Oxidase (Aox) which often plays a role in metabolic adaptability under stress (Edrich et al., 2024; Duvenage et al., 2019; Barsottini et al., 2020; Kido et al., 1797). Aox has been shown to be required for pathogenesis in *C. neoformans* and is not found in mammalian mitochondria (Szibor et al., 2022). Unfortunately, there are no current Aox inhibitors that have been shown to have high specificity, and those currently available, such as Salicylhydroxamic acid (SHAM), exhibit off target effects. In this work we sought to develop new Aox inhibitors with improved specificity. Previous work has demonstrated that the natural product Colletochlorin B is an effective inhibitor of the Aox from plant (Cortes et al., 2015), fungal (Battogtokh et al., 2018) and protozoan species (Ebiloma et al., 2018), with a typical IC_50_ in the 6–20 nm range *in vitro*. This includes microsporidian species, such as inhibition of the *Trachipleistophora hominis* lifecycle, but not inhibition of Aox-deficient *Encephalitozoon cuniculi* (Sendra et al., 2022). However, *in vitro* findings could not be replicated *in vivo*, indicating a need to improve the *in vivo* efficacy. The triphenyl-phosphonium cation (TPP^+^) has been shown to increase mitochondrial targeting of compounds (Cortes et al., 2015; Battogtokh et al., 2018; Ebiloma et al., 2018; Batheja et al., 2024; Cisneros et al., 2022; Meco-Navas et al., 2018), increasing mitochondrial accumulation up to 1,000-fold (Yousif et al., 2009). We describe the development and testing of TPP^+^ targeted derivatives of Colletochlorin B against *C. neoformans.*

Here we confirm that two mitochondrially-targeted Colletochlorin B compounds, ALTOX094 and ALTOX102, were able to significantly reduce *C. neoformans* growth and viability *in vivo*. However, although effective inhibitors of *C. neoformans,* our data suggested that the alkyl chain length used to link Colletochlorin to TPP^+^ conferred different modes of action. ALTOX094 promoted Aox independent membrane instability and necrosis. In contrast, ALTOX102 exhibited Aox specific effects and promoted loss of viability via an uncharacterized mechanism. Our findings suggest that targeting alkyl chains to mitochondria represents a promising approach to the development of new fungal specific inhibitors and as a new approach to tackle *C. neoformans* infection.

## Materials and methods

### Yeast strains and growth conditions

Wildtype (H99, Serotype A) and *Δaox1* deletion strains were generously gifted by Elizabeth Ballou (University of Exeter, United Kingdom) and were derived from the Madhani laboratory (University of California, San Francisco, California, United States, NIH funding R01AI100272) (Liu et al., 2008). Liquid cultures of wild-type (H99) and *Δaox1* strains were grown in YPD (1% Yeast extract (Difco), 2% Bactopeptone (Difco) and 2% Dextrose (Fisher Scientific)) on a 30°C or 37°C as stated on a rotary shaker at 180 revolutions per minute (rpm) in a sterile falcon tube. Culture plates for solid culture growth contained YPD media with added 20% Oxoid Technical Agar (Agar No. 3) and incubated at 30°C or 37°C where stated.

For automated assessment *C. neoformans* strains grown overnight at 30°C with shaking at 180 rpm and used to inoculate, either 24 well (1 mL), 48 well (500 μL) or 96 well (100 μL) at an OD_600_ of 0.1. Growth was measured as absorbance OD_600_ over 48 h using a BMG Labtech SPECTROstar Nano plate reader. Absorbance readings were collected and analysed by BMG Labtech MARS data analysis software. Growth rate, area under the curve (AUC), Minimum Inhibitory Concentration (MIC) and NIC were calculated using the Gompertz model for growth in GraphPad Prism doi: 10.

### Assessment of *C. neoformans* viability using colony forming unit (CFU) assays

YPD media was inoculated with a colony of the required *C. neoformans* strain and grown overnight at 30°C with shaking at 180 rpm. The next day, cells were inoculated to an OD_600_ of 0.1 in YPD containing drugs or solvent controls as indicated, and the culture was grown at 37°C, 180 rpm shaking for 2 h. Cells were then washed three times in Phosphate Buffered Saline (PBS) and diluted to 1×10^3^ cells per ml. 250 cells were then plated onto YPD agar plates and incubated at 37°C for 48 h and viable colonies were counted. Bar charts were constructed and significance tested using a Dunnett’s multiple comparisons test following a one-way ANOVA in GraphPad Prism doi: 10.

### Compound synthesis and purification

Analytical methodology details have been previously reported (McGown et al., 2022 #3)_._

ISSF31 - (E)-3-Bromo-5-(3,7-dimethylocta-2,6-dien-1-yl)-4,6-dihydroxy-2-methylbenzaldehyde.

**Table:** 

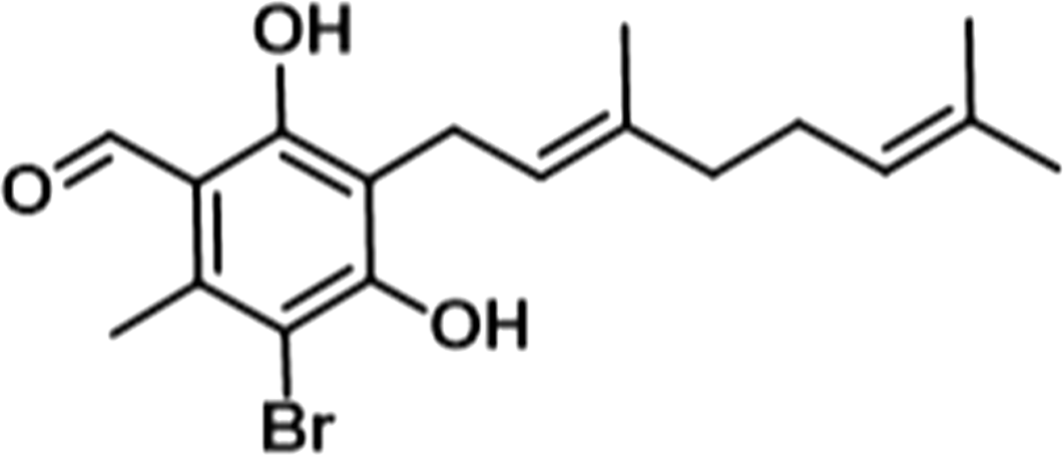

Potassium tert-butoxide (388.2 mg, 2.0 eq) and calcium chloride (134.4 mg, 0.7 eq) were suspended in methanol (10 mL) and cooled to −78°C then were added DMEDA (37 μL, 0.2 eq) and the reaction mixture was stirred for 5 min maintaining the same temperature. To the reaction mixture, 3-bromo-4,6-dihydroxy-2-methylbenzaldehyde (400 mg, 1.0 eq) was added before treatment with geranyl bromide (414 μL, 1.2 eq). The reaction was stirred at −78°C for 1 h before being allowed to warm to room temperature over 24 h.

Upon completion, the reaction mixture was concentrated to a residue, diluted with EtOAc (20 mL) and washed with water (20 mL) and brine (20 mL), dried over MgSO_4_ and concentrated to residue. The resulting mixture was purified by column chromatography (SiO_2_, 12 g, petroleum ether: ethyl acetate, 100:0 to 70:30 over 20 min) to yield ISSF31 as a colorless solid (66.0 mg, doi: 10.4%).

^1^H NMR (600 MHz, Chloroform-*d*) *δ* 12.72 (s, 1H), doi: 10.16 (s, 1H), 6.44 (s, 1H), 5.22 (t, J = 7.7 Hz, 1H), 5.05 (t, *J* = 7.2 Hz, 1H), 3.42 (d, *J* = 7.2 Hz, 2H), 2.64 (s, 3H), 2.06 (t, *J* = 7.6 Hz, 2H), 1.99 (t, *J* = 7.8 Hz, 2H), 1.79 (s, 3H), 1.65 (s, 3H), 1.57 (s, 3H). ^13^C NMR (151 MHz, Chloroform-*d*) *δ* 193.4, 162.7, 157.2, 139.6, 137.1, 131.5, 124.1, 124.0, 120.7, 114.4, 114.2, 106.0, 39.7, 26.6, 25.7, 22.2, 17.7, 17.6, 16.2. Calculated m/z [M + Na] C_18_H_23_BrNaO_3_ = 389.0728 and 391.0708. Experimental m/z [M + Na] C_18_H_23_BrNaO_3_ = 389.0711 and 391.0699 (ppm = +3.39) (Supplementary Figures S1, S2). LCMS RT 27.00 min A% = 94% [M-H] = 364.96 and 396.95. Minor impurity at 25.93 min with no detectable mass (Supplementary Figures S3, S4).

ALTOX094 - (12-(3-Bromo-5-formyl-2,6-dihydroxy-4-methylphenyl)dodecyl)triphenylphosphonium bromide.

**Table:** 

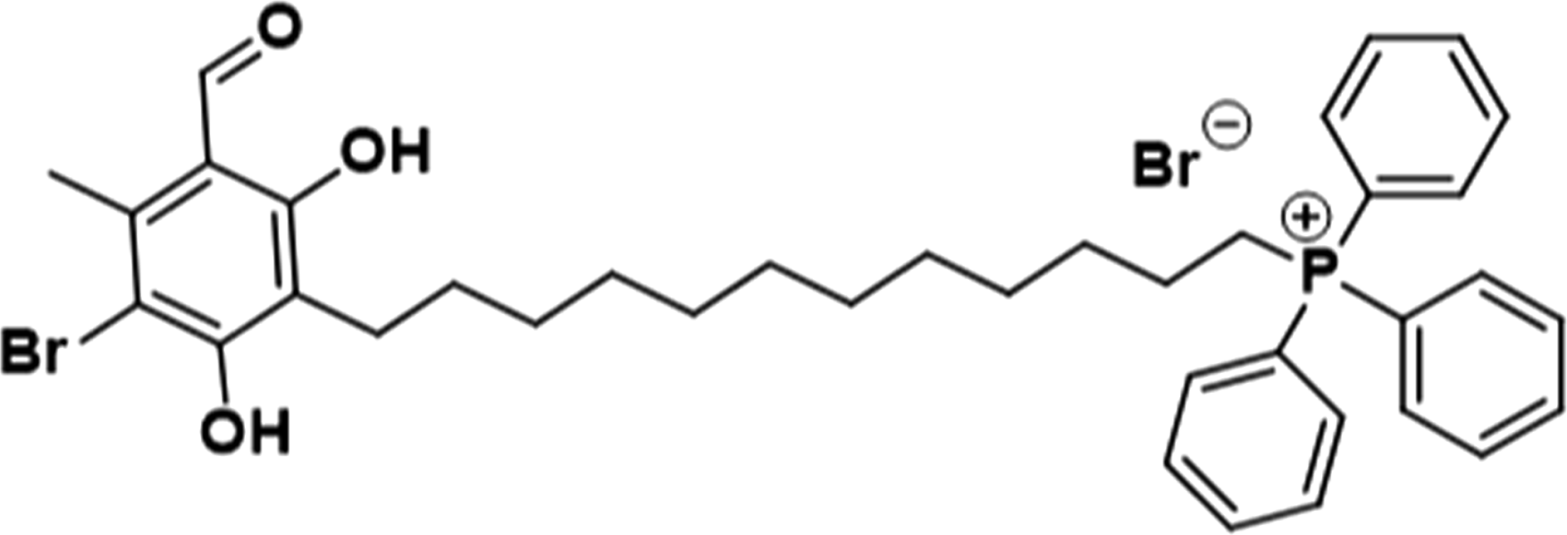

3-Bromo-4,6-dihydroxy-2-methylbenzaldehyde (500 mg, 1.0 eq) was dissolved in anhydrous THF (20 mL) with stirring under an inert atmosphere. The reaction mixture was treated with lithium metal (75.4 mg, 5.0 eq) and stirred at 40°C for 1 h. Next, (12-bromododecyl)triphenylphosphonium bromide (1281.2 mg, 1.0 eq) was added and the reaction mixture was stirred at 80°C for 72 h.

Upon completion, the reaction mixture was cooled to room temperature, concentrated to a residue, and resuspended in dichloromethane (DCM, 5 mL). The suspension was filtered through a Celite plug and the resulting filtrate was washed with water (10 mL) and brine (2 × 10 mL). The organics were dried over MgSO_4_ and concentrated to residue to give a pale brown, viscous oil.

The crude oil was purified by column chromatography (SiO_2_, 24 g, DCM: MeOH – 100:0 to 80:20 over 20 min) to yield an impure colorless oil, which was found to contain a minor persisting impurity (~4%) of (12-bromododecyl) triphenylphosphonium bromide. On a 25 mg scale this was further purified using mass directed assisted purification to yield ALTOX094 as a colorless solid (180.3 mg, 12.6%).

^1^H NMR (600 MHz, acetone-d_6_) *δ* doi: 10.27 (s, 1H), 8.51 (s, 1H), 7.98–7.90 (m, J = 8.3, 3.4 Hz, 9H), 7.81 (td, J = 8.3, 7.9, 3.3 Hz, 6H), 6.60 (s, 1H), 4.15 (t, J = 6.3 Hz, 2H), 3.64 (dt, J = 8.4, 5.4 Hz, 2H), 2.70 (s, 3H), 1.82 (p, J = 7.5 Hz, 2H), 1.77 (J = 7.5 Hz, 2H), 1.58 (p, J = 7.5 Hz, 2H), 1.52 (p, J = 7.5 Hz, 2H), 1.36 (dq, J = 15.2, 8.0, 7.4 Hz, 4H), 1.30–1.21 (m, 8H). ^13^C NMR (151 MHz, Chloroform-d) *δ* 193.3, 167.3, 165.3, 162.2, 142.5, 135.1 (d, J = 3.0 Hz) 133.5 (d, J = doi: 10.1 Hz) 130.6 (d, J = 12.7 Hz), 118.5 (d, J = 83.1 Hz), 113.9, 106.6, 98.9, 69.6, 30.5 (d, J = 15.8 Hz), 29.4, 29.2, 29.1 (d, J = 4.4 Hz), 28.6, 25.8, 22.6 (d, J = 4.4 Hz), 22.1, 17.9. Calculated m/z C_38_H_45_BrO_3_P^+^ [M + H] = 659.6393 and 661.2342. Experimental m/z C_38_H_45_BrO_3_P^+^ [M + H] = 659.2313 and 661.2325 (PPM = 3.5) LCMS RT 22.135 min A% = 85%* Mw = [M + H] 660.50 and [M-H] 659.25 (Supplementary Figures S5–S8).

*Single eluting peak. Please note – weak sample and peaks at 9.433 and 16.546 min are column artefacts.

ALTOX102 - (8-(3-Bromo-5-formyl-2,6-dihydroxy-4-methylphenyl)octyl)triphenylphosphonium bromide.

**Table:** 

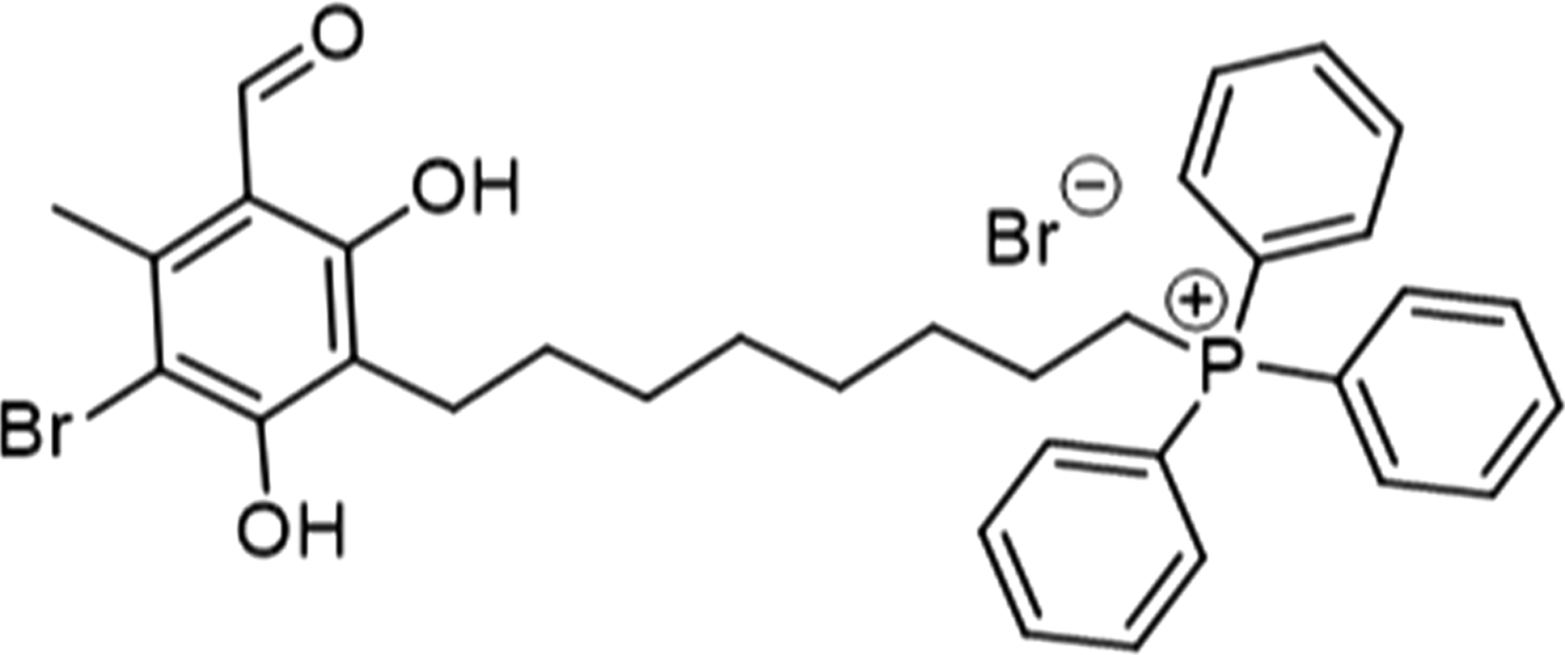

The same procedure was followed as reported in the synthesis of ALTOX094. ALTOX102 was isolated as a colorless solid (105.0 mg, 8%).

^1^H NMR (600 MHz, Chloroform-d) *δ* doi: 10.14 (s, 1H), 8.59 (s, 1H), 7.81–7.73 (m, J = 8.3, 3.4 Hz, 9H), 7.69 (td, J = 7.8, 3.4 Hz, 6H), 6.31 (s, 1H), 4.00 (t, J = 6.4 Hz, 2H), 3.58 (dt, J = 8.4, 5.4 Hz, 2H), 2.64 (s, 3H), 1.78 (p, J = 7.5 Hz, 2H), 1.60 (m, J = 5.6 Hz, 4H), 1.43 (p, J = 7.5 Hz, 2H), 1.31 (m, J = 5.5 Hz, 4H). ^13^C NMR (151 MHz, Chloroform-d) δ 193.3, 167.5, 165.3, 162.1, 142.5, 135.1 (d, J = 3.0 Hz) 133.6 (d, J = doi: 10.1 Hz) 130.5 (d, J = 12.4 Hz), 118.6 (d, J = 85.2 Hz), 113.9, 106.6, 99.0, 69.5, 30.4 (d, J = 15.9 Hz), 28.9, 28.8, 28.5, 25.7, 22.6 (d, J = 4.5 Hz), 22.0, 17.9. Calculated m/z C_34_H_37_BrO_3_P^+^ [M + H] = 603.1659 and 605.1638. Experimental m/z C_34_H_37_BrO_3_P^+^ [M + H] = 603.1690 and 605.1637 (PPM = 4.3) LCMS RT = 18.731 min, A% = 83%* [M + H] = 603.35 and 605.25 and [M-H] = 601.25 and 603.00 (Supplementary Figures S9–S12).

*Single eluting peak. Please note – weak sample and peaks at 5.357 and 9.700 min are baseline artefacts.

### High resolution respirometry

To determine oxygen consumption and capacity of *C. neoformans* strains, respirometry was performed using an O2k Oxygraph (Oroboros) high resolution respirometer. 5 mL of YPD was inoculated with a colony of required *C. neoformans* cultures and grown overnight at 30°C with shaking at 180 rpm. Cells were applied to the chambers of an Oroborus Oxygraph High Resolution Respirometer at a concentration of 1×10^6^ cells/ml. Routine respiration was recorded followed by the application of ALTOX102 or ALTOX094 at indicated concentrations, an equivalent DMSO solvent control was used in all cases for comparison. Statistics and respirometry graphs were recorded in Oroboros Datlab Version 4.2.1.62. Bar charts were generated and statistical significance was calculated using Dunnett’s multiple comparisons test following a one-way ANOVA in GraphPad Prism doi: 10.

### Flow cytometry for analysis of cell viability

Propidium Iodide (PI) staining was used to assess membrane integrity and 2′,7’-Dichlorodihydrofluorescein diacetate (H_2_DCF-DA) was used to detect hydrogen peroxide presence in *C. neoformans* cells. H99 and *Δaox1* mutant cells were grown overnight in 5 mL of YPD at 30°C with shaking at 180 rpm. Cells were harvested at indicated time points and resuspended in 300 μL of 100 μg/mL PI in sterile PBS or PBS containing 0.1 μM H_2_DCF-DA and incubated in the dark at 37°C for 10 min. Samples were then washed three times with sterile PBS and analysed by flow cytometry. Fluorescence intensity of cells was analysed using a BD Accuri™ C6 Plus Personal Flow Cytometer (BD Biosciences). Measurement events were gated on the flow cytometer to ensure that singlet yeast cells were being recorded. The flow cytometer was equipped with a Propidium iodide laser (488 nm, 585/40, 670 LP standard filter) and a FITC laser (488 nm, 533/30 standard filter). Approximately 15,000 events were collected per sample and the data was acquired and analysed using the BD Accuri C6 Plus software. Significance was calculated using Dunnett’s multiple comparisons test following a one-way ANOVA in GraphPad Prism doi: 10.

### Use of the *Galleria mellonella* infection model to assess *C. neoformans* virulence

A standardized *Galleria mellonella* infection model was used to assess *C. neoformans* virulence, as described previously ^23, 24^. *G. mellonella* larvae were ordered from Biosystems Technology (TruLarv™), which were prepared from a breeding colony without feedstuff antibiotic addition. Larvae were sterilised, uniformly aged and approximately 0.35 g. TruLarv™ were stored at 15°C until required. WT (H99) and *Δaox1 C. neoformans* were grown overnight in 5 mL of YPD at 30°C with shaking at 180 rpm. The next day, cells were harvested and TruLarv™ *G. mellonella* larvae were inoculated with 1 × 10^6^ CFU of wildtype (H99^−1^) or *Δaox1* mutant cultures (*Δaox1^−1^*). A 25 μL Hamilton syringe was used to inject 15 μL aliquots of either culture inoculum or a PBS control into the hemocoel of each larva via the last left proleg. Antifungal drugs (ALTOX094, ALTOX102) and appropriate solvent controls were injected using the same technique. For experiments that required multiple injections, such as those with both a cell culture inoculum and a drug inoculum, a different proleg was used for each injection, starting from the left last proleg and rotating left to right and moving proximally as needed. Larvae were then placed into sterile petri dishes containing filter paper and incubated at 37°C. Larvae were assessed every 24 h for 5 days and motility, melanization and survival using standardized scoring as described previously. A non-injection control set of larvae was also incubated. Larvae were considered dead if there was no motility response to touch. Kaplan-Meyer survival and melanization curves were plotted, and significance was calculated using a Log-Rank (Mantel-Cox) test in GraphPad Prism doi: 10. Each inoculum condition contained 10 larvae and was repeated independently three times unless stated otherwise.

### Haemolysis assay

A haemolysis assay was carried out using blood agar plates (1 g Peptone, 1 g Sodium Chloride (NaCl), 0.6 g Beef Extract, 3 g Technical Agar, 190 mL sterile mqH_2_O, 10 mL) defibrinated sheep blood (TCS Biosciences, ref.: SB054). Stock concentrations of the drugs were made at concentrations indicated and 20 μL of each drug was applied to sterile filter paper disks placed onto the agar and incubated at 37°C for 24 h. A PBS control and a positive 10% SDS control were also used. At 2 h and 24 h the zone of haemolysis (ZOH) was recorded, and results and significance was calculated using Dunnett’s multiple comparisons test following a one-way ANOVA in GraphPad Prism doi: 10.

### XTT assay for analysis of cytotoxicity

The Cell Proliferation Kit II (XTT) assay was carried out to determine ALTOX drug cytotoxicity on mammalian cells. The HEK293 mammalian cell line was seeded at a concentration of 10 × 104 cells/ well in 1 mL DMEM (Product No. D5671) containing 10% heat inactivated FBS (foetal bovine serum, Product No. 12106C) into microplates (tissue culture grade, 24 wells, flat bottom). Cell cultures were incubated for 24 h at 37°C and 5% CO_2_. The plate was then treated with ALTOX094 or ALTOX102 at the indicated concentrations for 2 h at 37°C and 5% CO_2_. After 2 h ALTOX drug treatment, 50 μL of the XTT labelling mixture was added to each well (25 mL XTT (sodium 3′-[1- (phenylaminocarbonyl)- 3,4- tetrazolium]-bis (4-methoxy6-nitro) benzene sulfonic acid hydrate)) at 1 mg/mL in RPMI 1640, 0.5 mL electron coupling reagent (PMS (N-methyl dibenzopyrazine methyl sulfate)) and incubated for 5 h at 37°C and 5% CO2. The absorbance of the samples was measured using a microplate reader at a wavelength of 492 nm and results and significance was calculated using Dunnett’s multiple comparisons test following a one-way ANOVA in GraphPad Prism doi: 10.

### Recombinant *C. albicans* Aox assay

Recombinant *C. albicans* Aox was expressed in *Escherichia coli* strain FN102 and the membranes were harvested as described previously ^25^. Dose response curves were generated using a Multiskan SkyHigh (Thermofisher) 96 well plate reader with the following conditions. Aox *E. coli* membrane was diluted to ~60 μg ml^−1^ in 65 mM 3-(N-morpholino) propanesulfonic acid (MOPS), pH 7.5 containing 1 mM KCN and 10 mM GMP and left to incubate with the inhibitor for 10 min (3-fold serial dilution). Reaction was initiated with the addition of NADH and followed at 340 nm for 10 min with readings taken every 8 s. Subsequent dose response curves were plotted in GraphPad Prism and significance was calculated using Dunnett’s multiple comparisons test following a one-way ANOVA.

## Results

### *In vitro* analysis of mitochondria targeted Colletochlorin B derivatives

**Figure 1:**
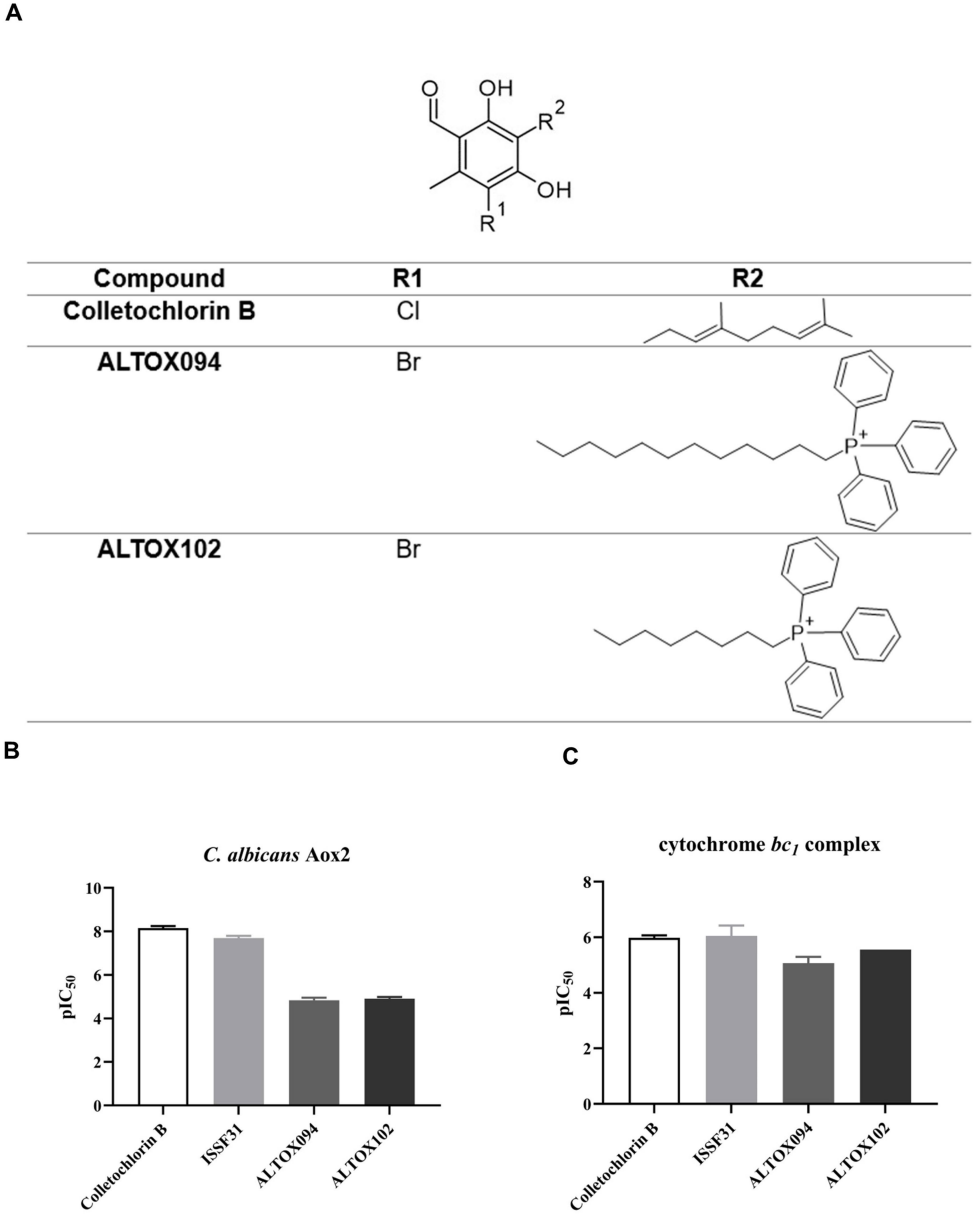
Inhibitory values for synthesised compounds against *C. albicans*. **(A)** Structures for natural product Colletochlorin B, and the ALTOX drugs synthesised for this study. Newly synthesized compounds were assessed for inhibitory activity against membrane bound recombinant Aox from *C. albicans* (rAox2) expressed in *E. coli* pIC_50_ values from inhibitor dose response curves against **(B)** *C. albicans* Aox2 and **(C)** *cytochrome bc_1_* complex. The IC50 values for each drug can be found in Table 2. All results plotted in triplicate ± SEM in GraphPad Prism. *n* = 3.

Our previous work determined that the natural product Colletochlorin B is a potent inhibitor of *C. albicans* Aox *in vitro* (Copsey et al., 2021), however as this inhibition was not observed *in vivo* we set out to improve the mitochondrial targeting of this compound. To this end, the isoprene tail was replaced by an alkyl chain of variable length (12-Carbon for ALTOX094 and 8-Carbon ALTOX102) capped with the TPP^+^ moiety. The chlorine found at R1 of Colletochlorin B was also replaced with bromine to yield ISSF31 partly, as bromine is larger than chlorine, and is more capable of forming halogen bonds (Wilcken et al., 2013) and less electronegative, to establish whether chlorine was critical for activity, also as it can be a useful synthetic handle for further coupling chemistry (Figure 1A). Our goal was to assess the effect of these Colletochlorin B derivatives against *C. neoformans* growth and viability. As a first step, however, we utilized an existing *in vitro* methodology (Young et al., 2020) to assess compounds ISSF31, ALTOX094 and ALTOX102 activity against membrane bound recombinant Aox from *C. albicans* (rAox2) (Figures 1B,C). As has been reported (Young et al., 2020) Colletochlorin B had a significant inhibitory effect on rAox2 (Figure 1B and Tables 1, 2). The substitution of chlorine for bromine (ISSF31) led to a small but significant reduction in efficacy (Figure 1B and Table 2). The addition of alkyl TPP^+^ (ALTOX094 and ALTOX102) however led to a reduction of approximately 50% in the observed inhibition of rAox2 *in vitro* (Figure 1B and Table 1). We also determined that Colletochlorin B, ISSF31, ALTOX094 and ALTOX102 were capable of inhibiting *C. albicans* cytochrome *bc*_1_ activity *in vitro*, albeit at a lower pIC50 than observed for rAox2 (Figure 1C and Table 1).

**Table 1:** pIC_50_ value table.

Compound	*C. albicans* Aox2	Cytochrome *bc_1_*
Colletochlorin B	8.2 ± 0.1	5.9 ± 0.05
Colletochlorin D	7.8 ± 0.02	4.3 ± 0.02
ISSF31	7.7 ± 0.1	6.0 ± 0.2
ISSF33	7.7 ± 0.1	4.4 ± 0.2
ALTOX094	4.8 ± 0.1	5.1 ± 0.1
ALTOX102	4.9 ± 0.1	5.4 ± 0.2

**Table 2:** IC_50_ value table.

Compound	*C. albicans* Aox2	Cytochrome *bc_1_*
Colletochlorin B	7.3 nM ± 2.4	1.1 μM ± 0.1
Colletochlorin D	15.9 nM ± 1.4	45.8 μM ± 2.2
ISSF31	21.1 nM ± 8.0	1.2 μM ± 0.6
ISSF33	19.6 nM ± 7.1	47.1 μM ± 1.7
ALTOX094	15.0 μM ± 1	9.1 μM ± 1.2
ALTOX102	11.1 μM ± 1	5.8 μM ± 1.4

### Efficacy of ALTOX094 and ALTOX102 against *C. neoformans*

**Figure 2:**
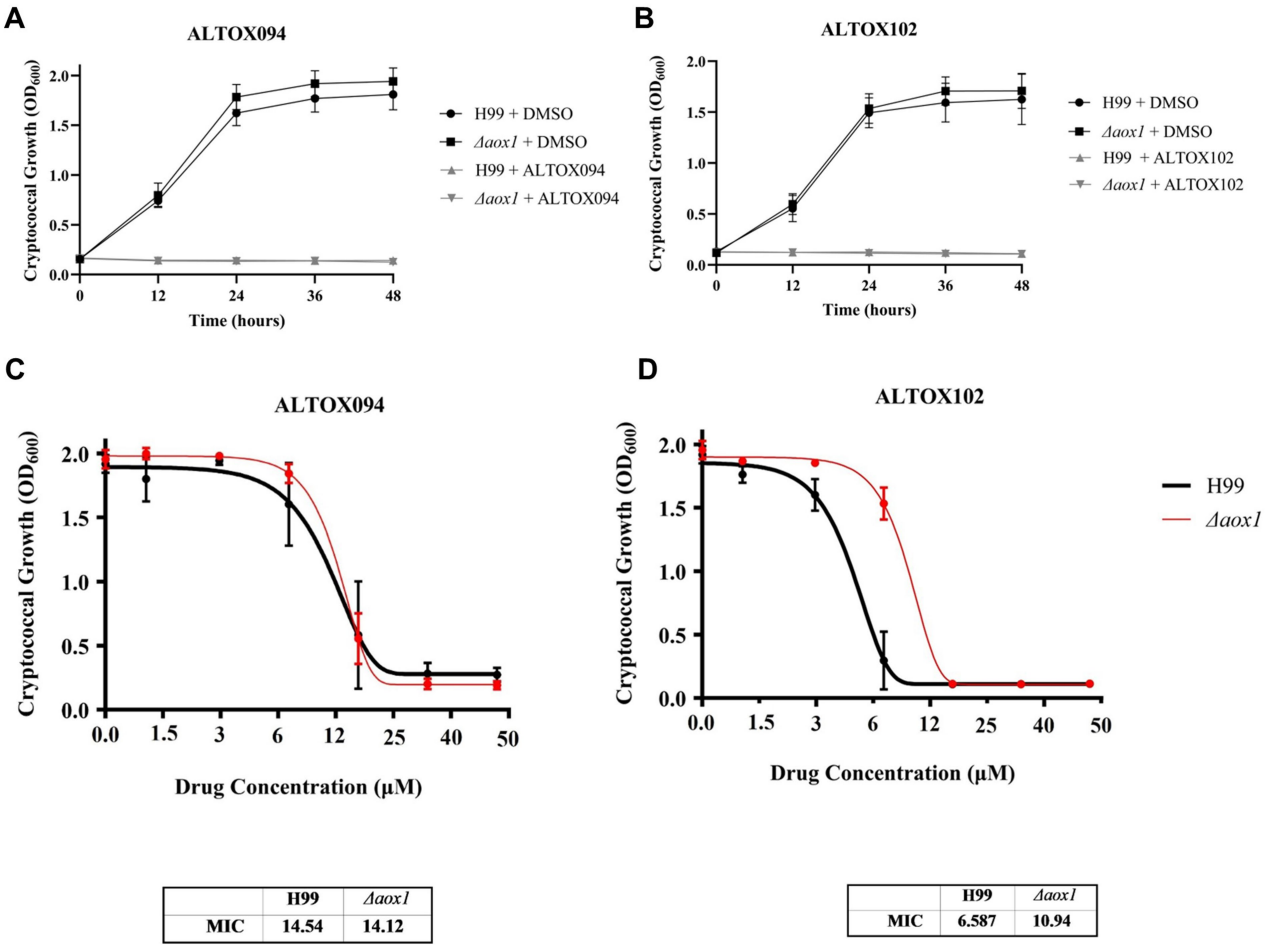
Screening of ALTOX compounds and their effect on *C. neoformans* growth. Representative example of wildtype (H99) and *Δaox1* null mutant (*Δaox1*) *C. neoformans* grown in YPD containing 100 μM **(A)** ALTOX094 or **(B)** ALTOX102 for 48 h at 37°C. Area Under Curve (AUC) values were calculated and plotted in GraphPad Prism. The Minimum Inhibitory Concentration (MIC90) values of wildtype (H99) and *Δaox1* null mutant (*Δaox1*) *C. neoformans treated with* **(C)** ALTOX094 and **(D)** ALTOX102 were calculated using the Gompertz equation for NIC/MIC90 determination in GraphPad Prism. Error bars represent ± SEM. Significance was calculated using Dunnett’s multiple comparisons test following a one-way ANOVA in GraphPad Prism. **** <0.0001, where *p* = 0.05. *n* = 9.

Given our *in vitro* findings that Colletochlorin B derivatives were capable of Aox and cytochrome *bc*_1_ inhibition, wildtype (H99) and *Δaox1 C. neoformans* cells were grown in the presence of either ALTOX094 or ALTOX102 at concentrations up to 200 μM. Growth curves were obtained (Figures 2A,B) and average Area Under Curve (AUC) values were determined to establish the Minimum Inhibitory Concentration (MIC90) of ALTOX094 and ALTOX102 (Figures 2C,D) using the Gompertz model for growth. MIC90 values for ALTOX094 were 14.5 μM for H99 and 14.1 μM for *Δaox1*, while for ALTOX102 the MIC90 values were 6.6 μM for H99 and doi: 10.9 μM for *Δaox1* mutant cultures, suggesting that ALTOX102 may have some specificity against Aox1 while the action of ALTOX094 is likely to be Aox1 independent (Figures 2C,D).

### Assessment of effects of ALTOX102 and ALTOX094 on *C. neoformans* respiration

**Figure 3:**
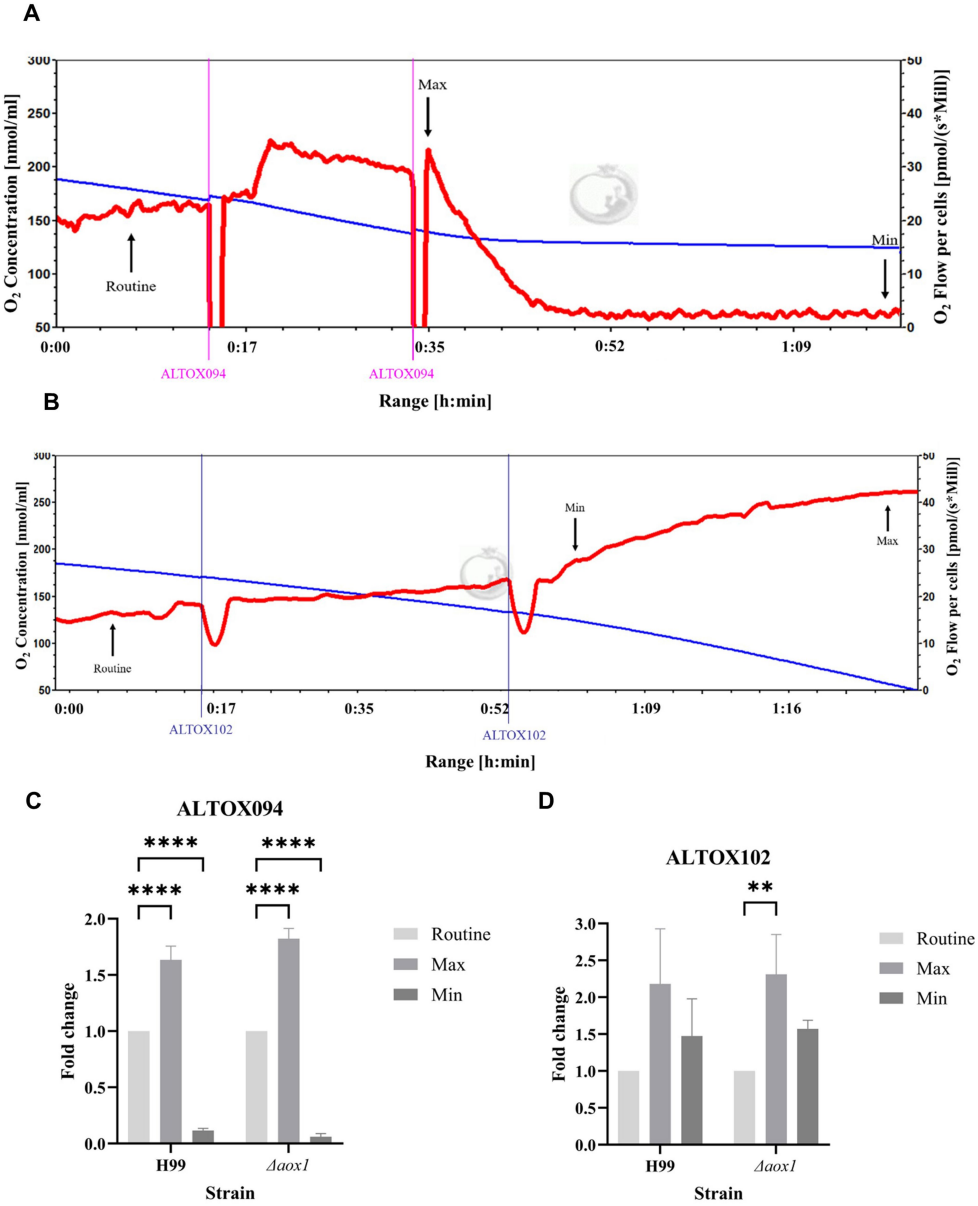
Respiratory profiles of *C. neoformans* exposed to ALTOX drugs. Representative example of respiration in H99 and *Δaox1* determined using HRR. Chambers were inoculated with 1 × 10^6^ cells after 24 h growth and treated with either ALTOX094 or ALTOX102 where indicated to a final concentration of the MIC90 for both drugs. **(A)** H99 + ALTOX094, **(B)** H99 + ALTOX102. Measurements of Maximum (Max) and Minimum (Min) respiration (O_2_ flow per cells) after drug exposure were taken and compared to the routine level of respiration (Routine) for each strain as indicated. **(C)** ALTOX094, **(D)** ALTOX102. Significance was calculated using Dunnett’s multiple comparisons test following a one-way ANOVA in GraphPad Prism. Error bars represent ± SD. * <0.05, ** <0.005, **** <0.0001, where *p* = 0.05. *n* = 3.

As ALTOX102 and ALTOX094 had strong inhibitory effects against *C. neoformans* growth (Figure 2) we investigated whether they also affected respiration in living cells. To achieve this, we conducted high resolution respirometry on wild type and *Δaox1 C. neoformans*. Interestingly addition of ALTOX094 showed a bi-phasic effect, whereby a lower MIC50 concentration (7.5 μM) induced an increase in respiration, but addition at the MIC90 level (15 μM) led to rapid loss of respiration in both H99 (Figure 3A) and *Δaox1* cells (Supplementary Figure S13). In contrast the addition of ALTOX102 after addition at the MIC90 concentration (11 μM) led to an increase in respiration in both H99 (Figure 3B) and *Δaox1* (Supplementary Figure S13). Measurements of Maximum and Minimum respiration after exposure to ALTOX094 (Figure 3C) and ALTOX102 (Figure 3D) were taken and compared to the routine level of respiration for each strain. These data showed that the effects of ALTOX094 and ALTOX102 were reproducible within living cells, with exposure to ALTOX094 reducing respiration by an average of 89% while ALTOX102 increased basal cellular respiratory activity by an average of 43.7%.

### ALTOX102 and ALTOX094 inhibit *C. neoformans* via different modes of action

**Figure 4:**
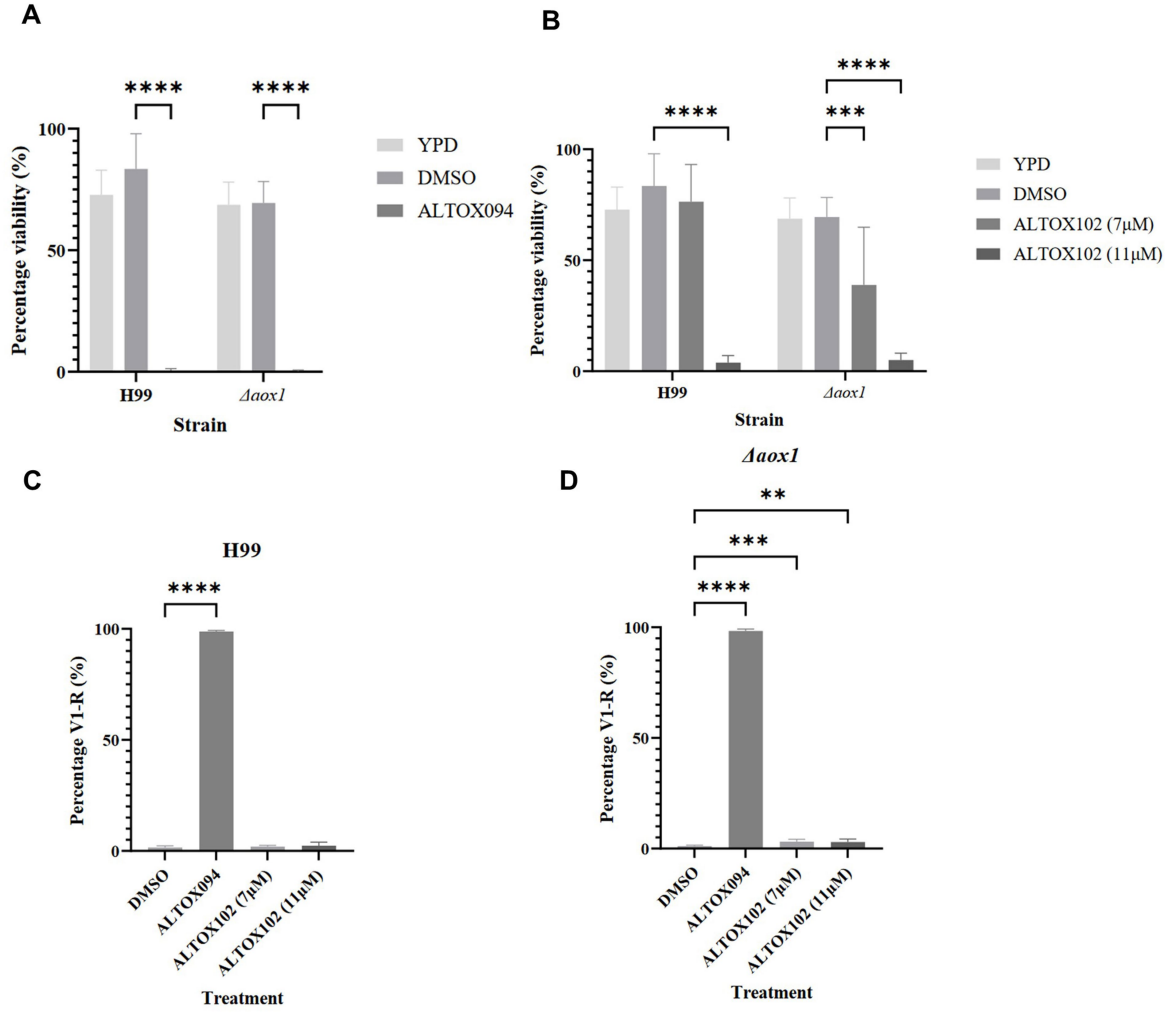
Viability assay of *C. neoformans* exposed to ALTOX094 and ALTOX102 treatment. The viability of wildtype (H99) and *Δaox1* null mutant (*Δaox1*) *C. neoformans* after a 2 h incubation in YPD containing ALTOX094 **(A)** and ALTOX102 **(B)** in comparison to a DMSO control. *C. neoformans* strains were stained with PI and analysed via Flow Cytometry after a 2 h incubation with ALTOX094 or ALTOX102 at the given MIC90. Percentage necrosis was measured via fluorescence in V1-R for H99 **(C)** and *Δaox1* **(D)** in comparison to a DMSO control. **(C)** Significance was calculated using Dunnett’s multiple comparisons test following a one-way ANOVA in GraphPad Prism. ** <0.005, *** <0.0005, **** <0.0001, where *p* = 0.05. Error bars represent ± SD. *n* = 9.

We wished to determine whether the inhibitory effects of ALTOX094 or ALTOX102 were accompanied by a loss of cell viability. The addition of either ALTOX094 or ALTOX102 at the MIC90 concentration for 2 h led to a significant loss of viability in both wildtype and *Δaox1* strains (Figures 4A,B). However, whilst the viability of *Δaox1 C. neoformans* was significantly reduced upon incubation with ALTOX102 at a concentration of 7.5 μM (MIC50), there was no significant inhibitory effect on wild-type H99 (Figure 4B), suggesting that cells lacking Aox1 were more sensitive. To determine the mode of viability loss *C. neoformans* were treated with ALTOX094 or ALTOX102 at the given MIC90 value for 2 h and assessed for Propidium Iodide (PI) uptake, a marker of necrosis. Exposure to ALTOX094 induced necrosis in 99% of wild type (Figure 4C) and 98% of *Δaox1* mutant *C. neoformans* cells (Figure 4D). In contrast, treatment with 7 μM or 11 μM ALTOX102 for 2 h resulted in minimal necrosis, with only 2% of wildtype (Figure 4C) and 3% of *Δaox1* (Figure 4C) showing PI uptake. The effects of ALTOX094 or ALTOX102 on viability did not correlate with an increase in ROS levels, as assessed by 2′,7’-Dichlorodihydrofluorescein diacetate (H_2_DCF-DA) staining in wild type or *Δaox1 C. neoformans* cells (Supplementary Figure S14), suggesting that oxidative stress is not involved in the loss of viability.

### Effects of ALTOX drug application on *C. neoformans* virulence

**Figure 5:**
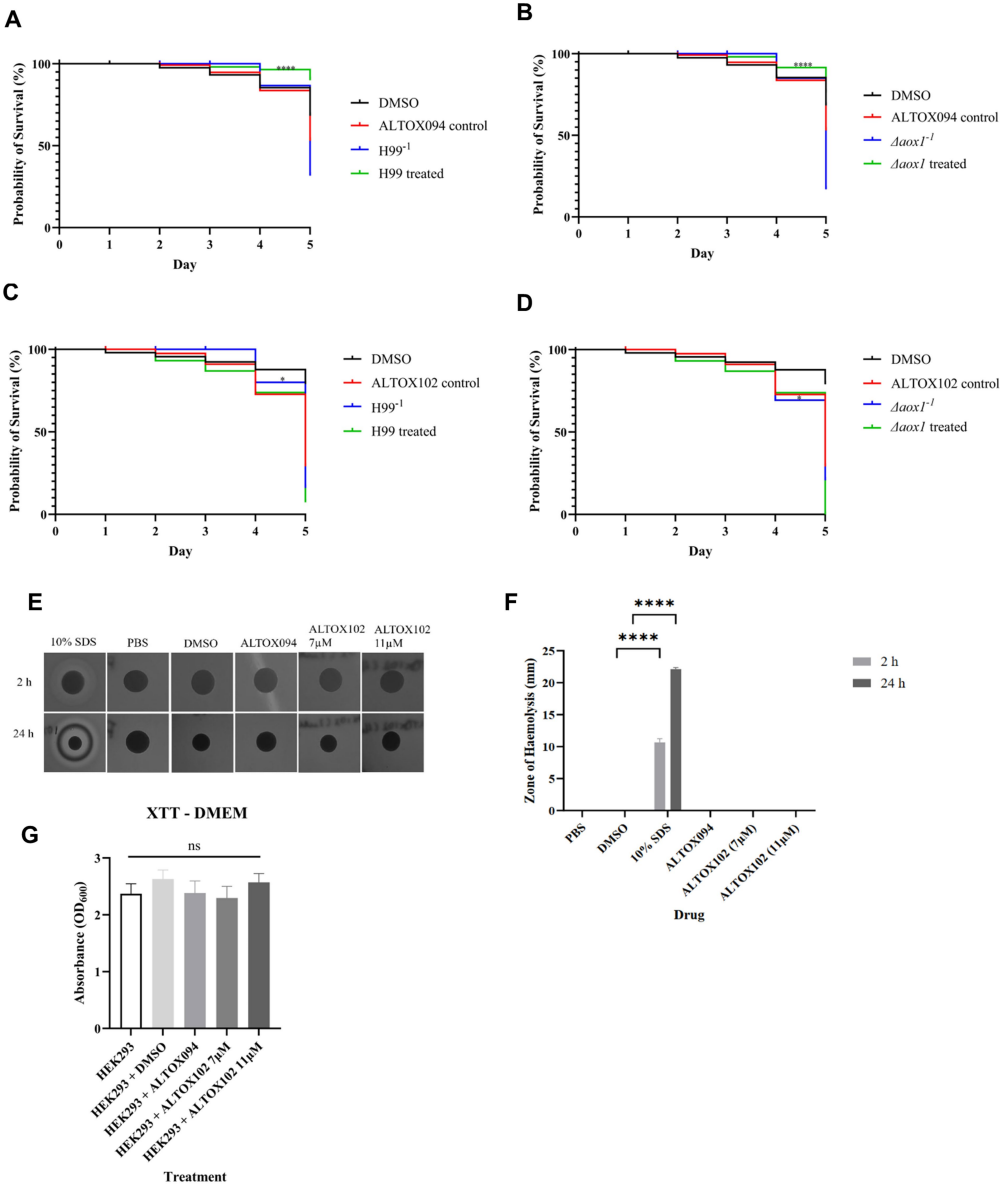
Mortality of *Galleria mellonella* exposed to ALTOX drugs. TruLarv™ G. mellonella larvae were infected with 1 × 10^6^ CFU *C. neoformans* that were pre-treated with either ALTOX094 (*n* = 30) or ALTOX102 (n = 10) at the MIC90 for 2 h before incubation at 37°C for 5 days. Larvae were injected through the lower left proleg with either **(A)** H99/ALTOX094, **(B)** *Δaox1*/ALTOX094, **(C)** H99/ALTOX102, or **(D)** *Δaox1/*ALTOX102 and compared to a 30% DMSO control. Kaplan-Meyer curves were plotted, and significance was calculated using a Log-Rank (Mantel-Cox) test in GraphPad Prism. **(E)** ALTOX094 and ALTOX102 were administered to filter paper disks on agar plates containing 5% sheep blood at the MIC90 and incubated at 37°C. **(F)** The zone of haemolysis (ZOH) was measured after 2 h exposure and 24 h exposure. **(G)** Mammalian cell line HEK293 was exposed to ALTOX094 or ALTOX102 at the MIC90 for 2 h at 37°C and 5% CO_2_ compared to a DMSO control and an XTT cytotoxicity assay was carried out for 5 h at 37°C and 5% CO_2_. Significance was calculated using Dunnett’s multiple comparisons test following a one-way ANOVA in GraphPad Prism * <0.05, ** < 0.005, *** <0.0010, **** < 0.0001 where *p* = 0.05.

To investigate whether the action of ALTOX094 and ALTOX102 reduced virulence and displayed fungal specificity we made use of *Galleria mellonella* infection model and haemolysis assays. TruLarv™ *G. mellonella* larvae were inoculated with 1 × 10^6^ CFU of wildtype (H99^−1^) and *Δaox1* mutant cultures (*Δaox1^−1^*), followed by injection of MIC90 15 μM ALTOX094 or MIC90 11 μM ALTOX102 on day 1 or an injection control. No significant effect on *G. mellonella* viability was observed over the 5-day time course following injection with the MIC90 15 μM ALTOX094 (Figures 5A,B), however treatment with ALTOX102 did increase *Galleria* mortality by day 5 (Figures 5C,D). In contrast to a previous report (Shamima et al., 2003) we did not observe a significant difference between H99 and *Δaox1* virulence in *the G. mellonella* infection model at the infection titres used (Figure 5). However, in line with its effects on cell viability pre-treatment of wildtype or *Δaox1* mutant cultures cells with ALTOX094 for 2 h at before injection significantly decreased larval mortality (Figure 5B). The addition of ALTOX102 at the MIC90 11 μM significantly increased mortality by day 5 (Figures 5C,D) and shows an increased toxicity to *Galleria* larvae when injected after infection with *Δaox1* (Figure 5D).

To determine if ALTOX094 and ALTOX102 had detrimental effects on lipid bilayer integrity we conducted a haemolysis assay. Each compound was spotted onto agar plates containing 5% sheep’s blood up to ten times the MIC90 value, with SDS used as positive control, and a zone of haemolysis (ZOH) was measured at 2 h and 24 h. Alpha-haemolysis (red blood cell damage) was evident by a green-tinge and beta-haemolysis (complete lysis) led to a zone of clearance (Buxton, 2005). Neither ALTOX094 or ALTOX102 showed any sign of haemolysis in comparison to the SDS positive control (Figures 5E,F). No significant haemolytic activity was seen from either ALTOX094 or ALTOX102, even when the concentrations ranged at x2, x5 and x10 of the highest MIC90 of each drug (Supplementary Figure S15).

To determine whether ALTOX094 and ALTOX102 were cytotoxic to mammalian cell lines, an XTT assay was carried out. HEK293 cells were seeded into a 24-well plate containing DMEM plus 10% foetal bovine serum (FBS), and each compound and a DMSO control was added to the plate at the MIC90 for 2 h at 37°C and 5% CO_2_. Following incubation, addition of the XTT reagent determined that both ALTOX094 and ALTOX102 had no significant effect on mammalian cell metabolism and were not cytotoxic to the HEK293 cell line (Figure 5G).

### Determining the active moiety of ALTOX094 and ALTOX102

ALTOX094 and ALTOX102 exposure leads to a rapid loss of *C. neoformans* viability, however despite differing only in alkyl chain length our data suggest the compounds elicit distinct effects on the cell. We therefore conducted a structure function analysis to determine the role of alkyl chain length, bromination and presence of the pharmacophore groups on *C. neoformans* inhibition. Reference compounds relating to ALTOX094 (Decyl TPP^+^, Dodecyl TPP^+^, TPPC3 Supplementary Table S1) and ALTOX102 (Octyl TPP^+^ Supplementary Table S2) were tested. As we suspected that membrane disruption may play an important role in the effects of ALTOX094 and ALTOX102 we used Mono-Alkyl-Lipophilic Cation (MALC) compounds, which are cationic surfactants that have reported antifungal activity through alteration of plasma membrane permeability (Steinberg et al., 2020), as a comparator. We tested the activity of two Mono-alkyl lipophilic cation (MALC) compounds with different chain lengths, an 18 Carbon (MALC-18STAB) and a 10 Carbon (MALC-10STAB). These experiments were included to assess whether the effects of MALC compounds on viability and membrane permeability were also dependent on alkyl chain length. We also included base molecules for both drugs (TPP^+^, ISSF31) in our analysis as further controls. The compounds tested and summaries of findings are set out within Supplementary Tables S1, S2.

**Figure 6:**
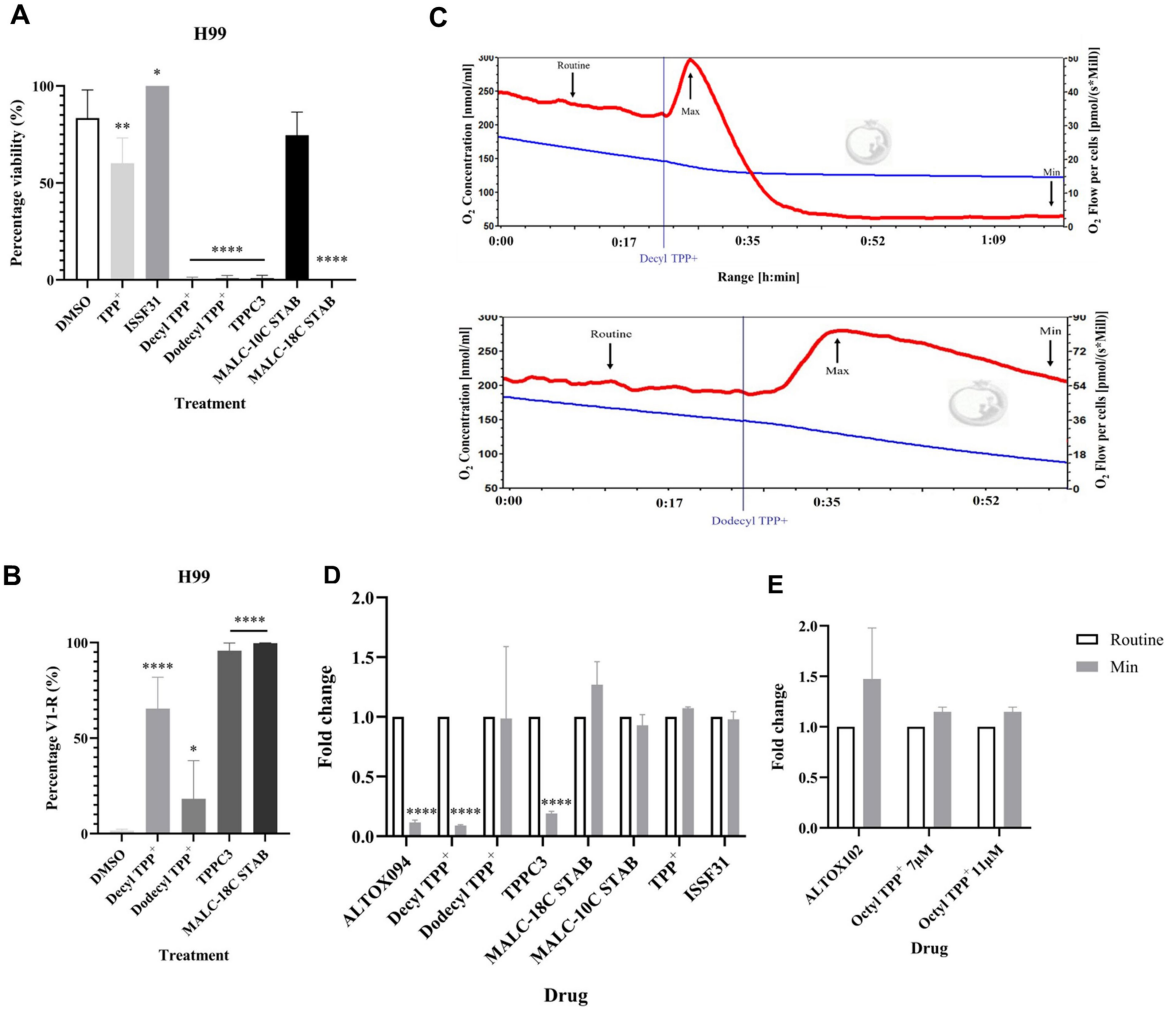
Screening of ALTOX094 reference compounds and their effect on *C. neoformans* growth, viability and respiration. **(A)** The viability of wildtype (H99) cells exposed to TPP+ and ISSF331, and ALTOX094 reference compounds Decyl TPP+, Dodecyl TPP+, TPPC3, and MALC reference compounds MALC-10C STAB and MALC-18C STAB. Cultures were grown in YPD containing each compound at the MIC90 for 48 h at 37°C. **(B)** Wildtype (H99) *C. neoformans* was stained with PI and analysed via Flow Cytometry after a 2 h incubation with ALTOX094 reference compounds. Percentage necrosis was measured via fluorescence in V1-R in comparison to a DMSO control. **(C)** Representative example of respiration in H99 determined using HRR. Chambers were inoculated with 1 × 106 cells after 24 h growth and treated with either Decyl TPP+ or Dodecyl TPP+ where indicated at the MIC90 for both drugs. Measurements of Minimum (Min) respiration (O2 flow per cells) after **(D)** ALTOX094 and **(E)** ALTOX102 reference drug exposure was taken and compared to the routine level of respiration (Routine) as indicated (n = 3). Significance was calculated using Dunnett’s multiple comparisons test following a one-way ANOVA in GraphPad Prism. * <0.05, ** < 0.005, *** <0.0005, **** <0.0001, where *p* = 0.05. Error bars represent ± SEM. *n* = 9.

Treatment with ALTOX094 reference compounds Decyl TPP^+^, Dodecyl TPP^+^, TPPC3 and MALC-18C STAB significantly reduced viability of both H99 and *Δaox1* cells (Figure 6A and Supplementary Figure S16A). Interestingly treatment with TPP^+^ led to a small decrease in viability but ISSF31 and MALC-10C STAB did not decrease viability in H99 or *Δaox1* cells (Figure 6A and Supplementary Figure S16A). The loss of viability was linked to a necrotic fate upon treatment with Decyl TPP^+^, TPPC3 and MALC-18C STAB, with necrosis present but at a significantly lower level in in the case of Dodecyl TPP^+^ (Figure 6B and Supplementary Figure S16B). The reference compounds were also tested for their ability to affect respiration. Addition of TPP^+^, ISSF31 and MALC-10C STAB did not influence respiration. However, Decyl TPP^+^ and TPPC3 mimicked the effect observed upon ALTOX094 increasing in the rate of respiration upon addition followed by a rapid decline (Figures 6C,D). Dodecyl TPP^+^ showed a similar rapid increase in respiration but a slower decline to that returned to a routine respiration rate (Figures 6C,D).

**Figure 7:**
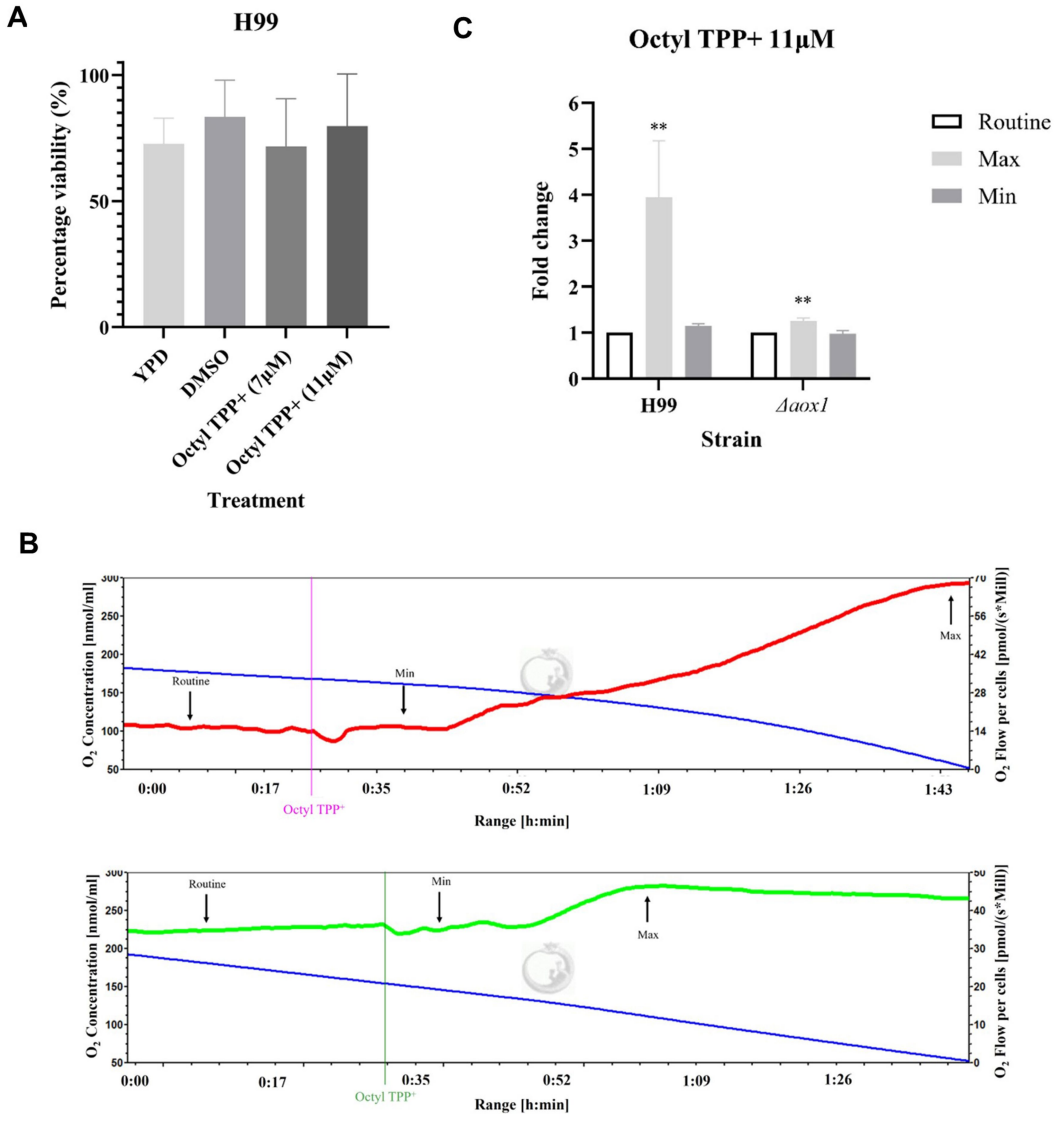
Screening of ALTOX0102 reference compounds and their effect on *C. neoformans* growth, viability and respiration. **(A)** The viability of wildtype (H99) *C. neoformans* after a 2 h incubation in YPD containing ALTOX102 reference compound Octyl TPP^+^ at the MIC90 of 7 μM and 11 μM, in comparison to a DMSO control. **(B)** Representative example of respiration in H99 (red) and Δaox1 mutant cells (green) determined using HRR. Chambers were inoculated with 1 × 10^6^ cells after 24 h growth and treated with 11 μM Octyl TPP^+^. Measurements of Maximum (Max) and Minimum (Min) respiration (O_2_ flow per cells) after drug exposure were taken and compared to the routine level of respiration (Routine) as indicated for Octyl TPP+ **(C)**. Significance was calculated using Dunnett’s multiple comparisons test following a one-way ANOVA in GraphPad Prism. ** < 0.005, *** <0.0005, **** <0.0001, where p = 0.05. Error bars represent ± SEM. n = 9.

Viability and respiration assays were repeated using the ALTOX102 reference compound Octyl TPP^+^ at the MIC90 of both 7 μM and 11 μM. In contrast to the ALTOX094 control compounds Octyl TPP^+^ treatment did not decrease viability (Figure 7A). Interestingly, the addition of Octyl TPP^+^ led to an increase in respiration that was not apparent in cells lacking Aox1, suggesting an Aox1 dependent response (Figures 7B,C).

## Discussion

The initial aims of this study were to enhance the efficacy of Colletochlorin B as a mitochondrial electron chain inhibitor in fungal cells. This was a logical extension of initial *in vitro* findings that Colletochlorin B may be able to inhibit both fungal cytochrome *bc1* and Aox. The addition of a TPP^+^ moiety and alkyl chain linker were made to increase uptake and mitochondrial targeting to enhance efficacy *in vitro*. In contrast to the reported results of Colletochlorin B the derivatives ALTOX094 and ALTOX102 were effective in preventing *C. neoformans* cell growth and induced a loss of viability. While ALTOX094 induced high levels of necrosis, ALTOX102 appeared to increase respiration suggesting that these compounds, that differ in only their alkyl chain lengths, have distinct modes of action against *C. neoformans*. One possibility is that ALTOX094 acts in the same way as a proton ionophore, such as FCCP, whereby it uncouples respiration leading to an initial increase in respiration but that its action is also disruptive to mitochondrial membranes leading to a loss of substrate supply and hence a steep decline in respiration, as was observed. However, our finding that ALTOX094 promotes rapid necrosis suggests that it may have a broader role in membrane destabilization as would be displayed by a surfactant. This hypothesis is supported by the finding that MALC compounds, which have surfactant and anti-fungal properties, showed a similar effect on respiration and necrotic cell death. It is interesting to note that ALTOX094 does appear to have fungal specificity as lytic effects on red blood cells or toxicity to *Galleria mellonella* were not observed. Our findings suggest that the Colletochlorin B moiety is not required for the effects of ALTOX094 on cell viability, but its effects are a consequence of alkyl chain length and targeting to the mitochondria. While a TPP^+^ Octyl compound had no effect on cell viability we observed a robust necrotic cell death when this was extended to Decyl or Dodecyl forms, suggesting that alkyl chain length is critical.

In contrast to the necrotic effects of ALTOX094, the reduction in growth and viability observed upon ALTOX102 treatment did not occur because of necrosis or inhibition of respiration. We did observe that cells lacking Aox1 were more sensitive to ALTOX102, which may suggest that Aox1 is required for resistance. One possibility is that ALTOX102 inhibits the cytochrome *bc*_1_ complex, which in turn induces Aox1 and an increase in respiration. However, we did not observe a difference in respiration when cells lacking Aox1 were challenged with ALTOX102. This may suggest the presence of another uncharacterized oxidase within *C. neoformans*, as has been noted in *C. albicans* and *C. parapsilosis* (Duvenage et al., 2019; Milani et al., 2001; Guedouari et al., 2014; Helmerhorst et al., 2005). An alternative explanation is that ALTOX102 induces an increase in oxygen consumption directly by an uncharacterized effect, which may prove deleterious to cell viability. However, we did not observe an increase in ROS in wild type or cells lacking Aox1 upon ALTOX102 exposure, which may be expected to occur when electron transport chain activity is rapidly increased. ROS production and regulation in response to mitochondrial uncouplers is debated (Demine et al., 2019; Tahara et al., 2009; Shabalina et al., 2014; Shabalina and Nedergaard, 2011), with some models confirming a decrease in ROS production following induction of mitochondrial uncoupling (Demine et al., 2019). Interestingly, while the respiratory traces of Octyl TPP^+^ mimicked the respiratory increase observed after ALTOX102 addition in wildtype cells, this did not confer a depletion in cell viability and no respiratory response or reduction in viability was observed in *Δaox1* mutant cells. Such a result suggests that the fungicidal action of ALTOX102 is dependent on the presence of Colletochlorin B, although the mode of action remains in question. It may be that ALTOX102 does target the electron transport chain and that the increase in respiration leads to a loss of cellular homeostasis that is sufficient to lead to a loss of viability loss of viability in *C. neoformans*. We cannot discount the possibility that the effects of ALTOX102 on viability may occur via alternative targets and this will require further investigation.

Overall, our data suggest that alkyl chains of specific lengths coupled to a TPP^+^ moiety show promise as useful anti-fungal compounds. Our assessment of ALTOX094 control compounds suggest that both Decyl TPP^+^ and TPPC3 mimic ALTOX094, suggesting that effects on viability and membrane disruption were a result of TPP^+^ targeted alkyl chains alone. Dodecyl TPP^+^ was not as effective as TPPC3, which differs only in the presence of a bromine, in fungicidal activity. Although the reason behind this difference is unclear, one possibility is that the bromine group increases the stability and cytotoxicity of TPPC3 within cells. Alternatively, lack of bromine may impart altered lipophilic properties, such as reduced bioavailability of Dodecyl TPP^+^ or alternative targeting. Interestingly, studies have cited Dodecyl TPP^+^ activity as effective against cancer stem cells, where it was shown to inhibit mitochondrial oxygen consumption rate (OCR) and to shift metabolism towards glycolysis (De Francesco et al., 2019). Decyl TPP^+^ has also been shown to alter OCR, ATP generation and mitochondrial membrane potential following treatment of human skin fibroblasts (Bulthuis et al., 2022). It may be that longer alkyl chains, such as Dodecyl TPP^+^, require the influence of a stabilizing end group while shorter compounds such as Decyl TPP^+^ do not. Our findings add to growing evidence that functionalised alkyl chains, such as alkyl gallates, demonstrate useful disruptive effects against fungal membranes, showing promising activity against pathogenic fungi and antibiofilm activity. We suggest that functionalized alkyl chains may be developed further as an effective antifungal class against fungal pathogens such as *C. neoformans.*

## Data Availability

The original contributions presented in the study are included in the article/Supplementary material, further inquiries can be directed to the corresponding authors.
