# Supplementary material for: Novel mitochondrial-targeted alkyl chains act as fungal specific inhibitors of C. neoformans
Source: Front Microbiol. 2025 Feb 4;15:1505308. doi: 10.3389/fmicb.2024.1505308 (PMC11832467; doi:10.3389/fmicb.2024.1505308)
Supplement: Supplementary file 1 [file Data_Sheet_1.pdf]

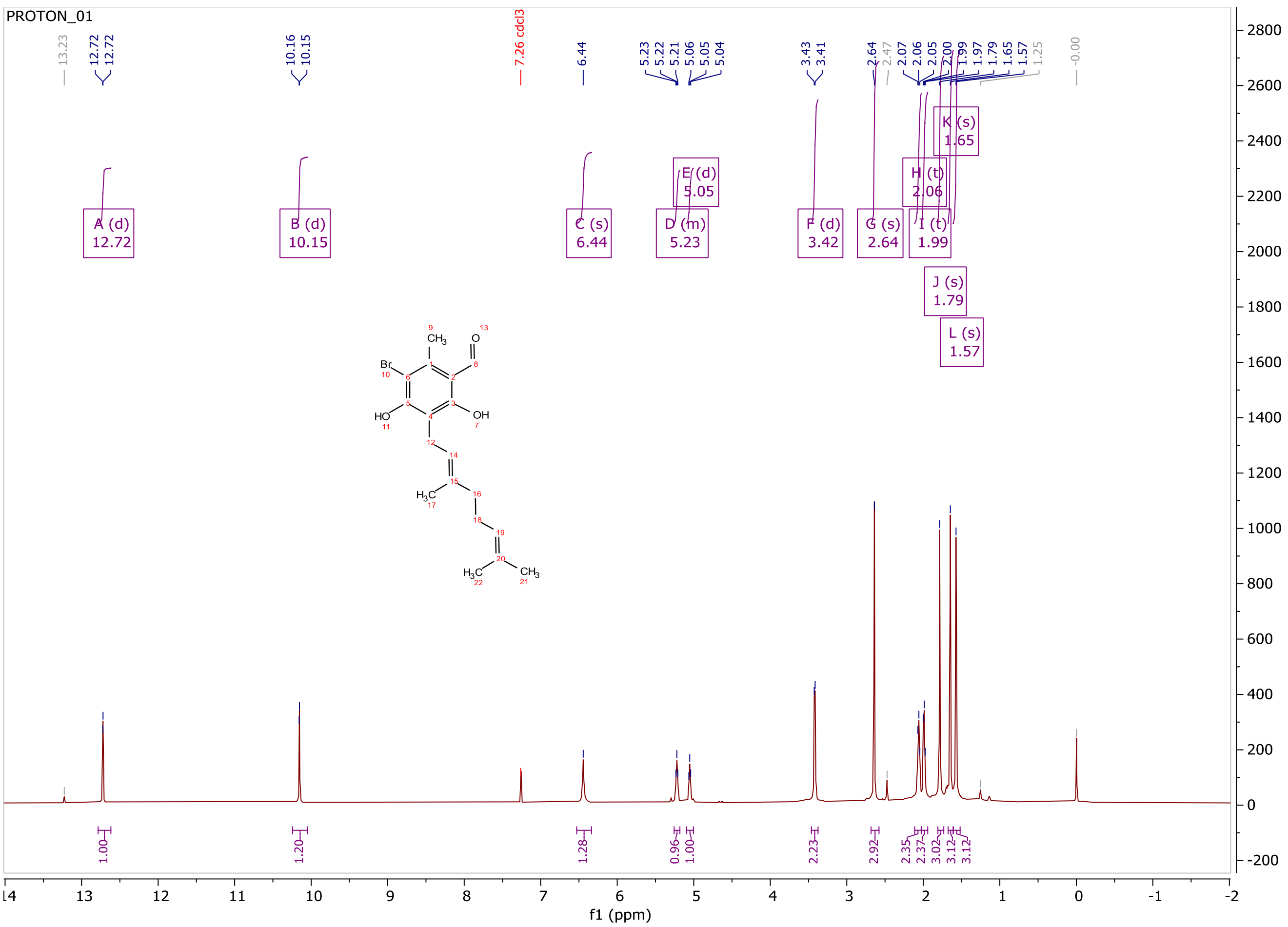

Supplementary Figure S1: ^{1}H NMR spectra of ISSF31.

^{1}H NMR spectra of final published compound ISSF31 in chloroform-d recorded on a Varian 600 MHz spectrometer. Data reported in ppm referenced to TMS found to be in concordance with reported structure.

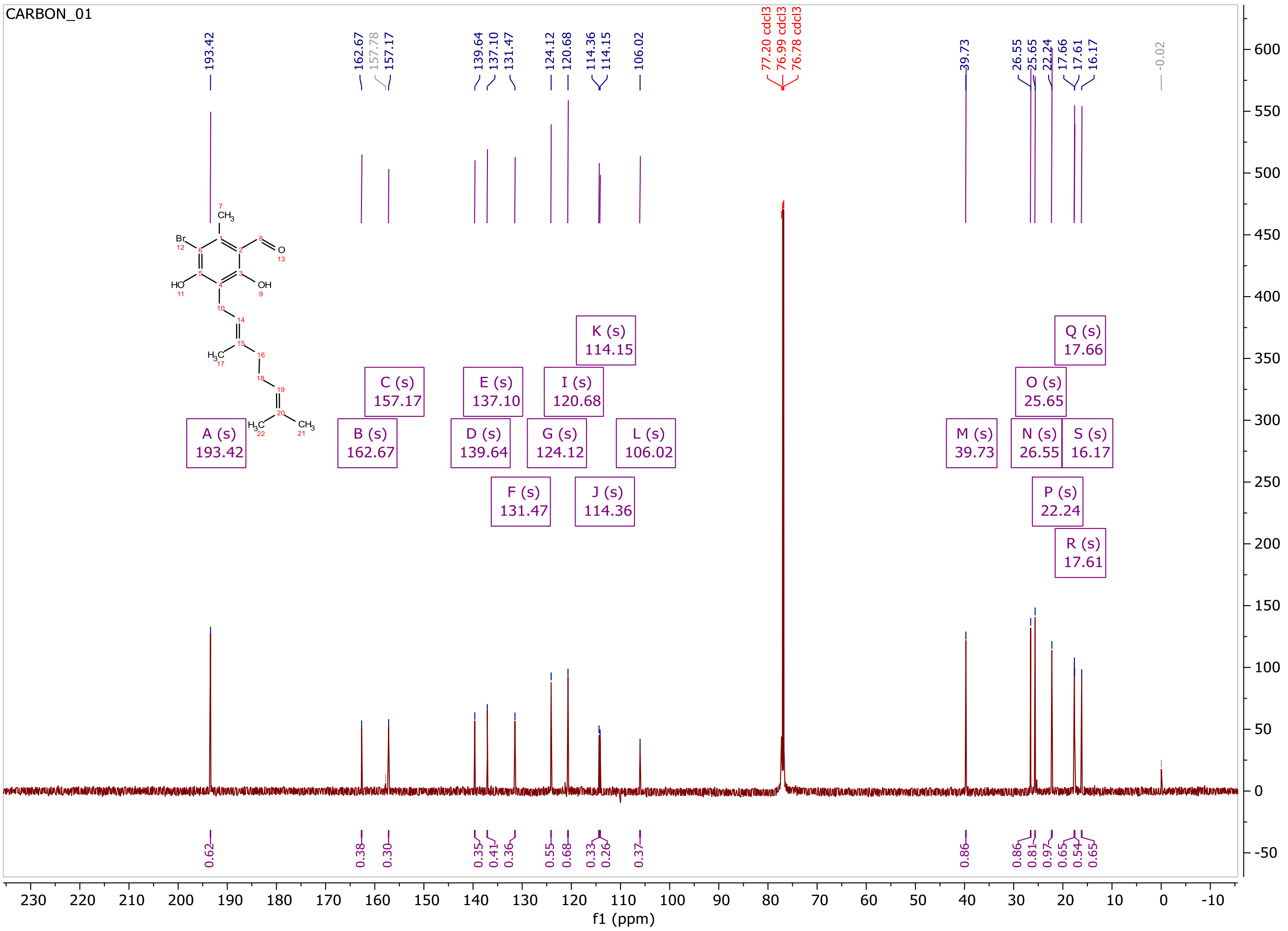

Supplementary Figure S2: ^{13}C NMR spectra of ISSF31.

^{13}C NMR spectra of final published compound ISSF31 in chloroform-d recorded on a Varian 600 MHz spectrometer. Data reported in ppm referenced to TMS found to be in concordance with reported structure.

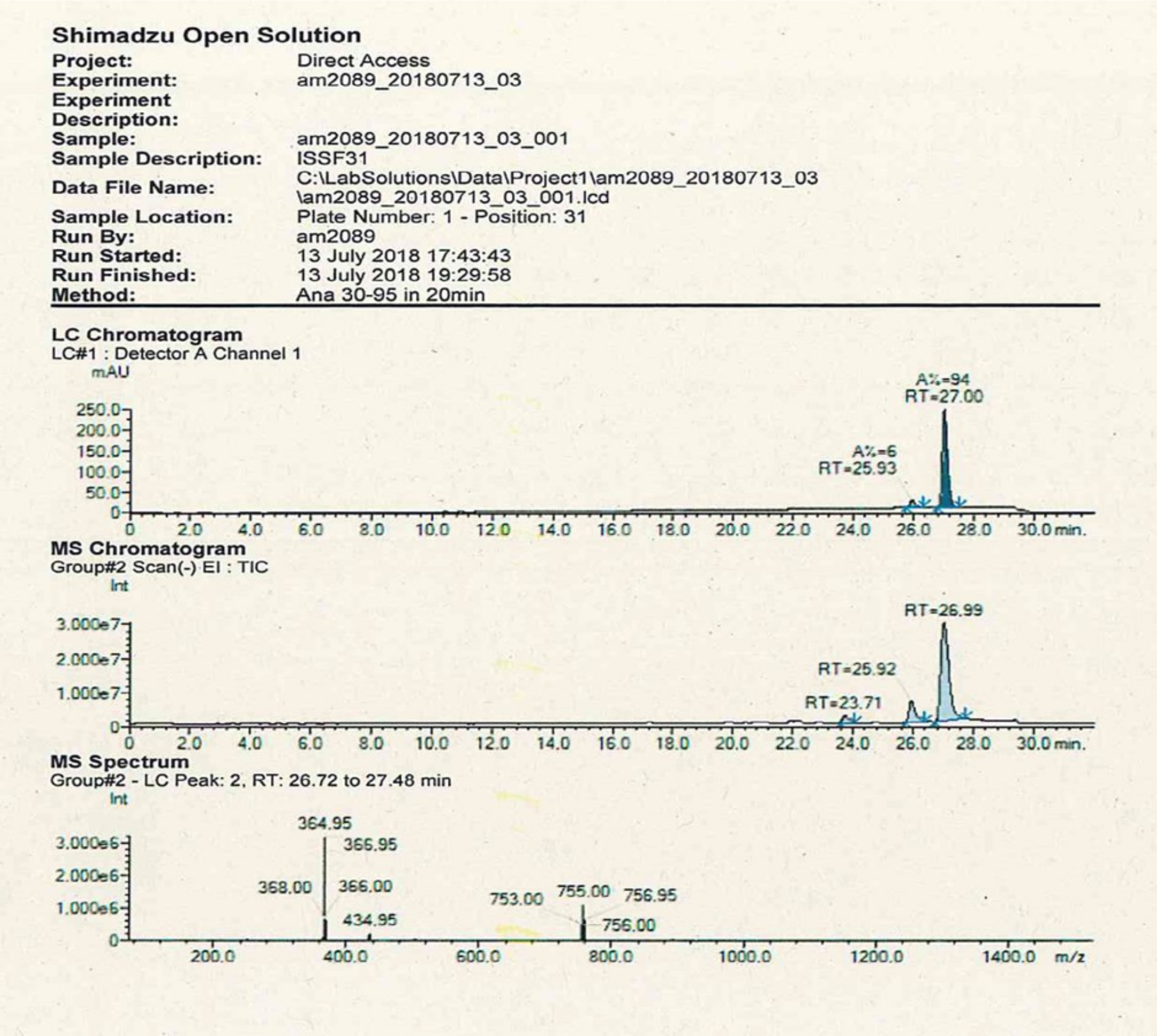

Supplementary Figure S3: LCMS purity of ISSF31.

LCMS analysis of reported compound ISSF31 run on a Shimadzu LCMS-2020 system equipped with a Gemini® C18 110 Å column and a UV detector set at 254 nm. Percentage purity measurements were run using a 30 minute method in water/acetonitrile both modified with 0.1% formic acid (5 minutes 95/5, 5/95 over 20 minutes, 5 minutes 5/95). Experimental data in concordance with reported structure.

Generic Display Report

Analysis Info

Analysis Name	D:\Data\Alinanopos\ANDREW_9893_000001.d	Acquisition Date	11/06/2018 14:53:27
Method	pos20090608esi	Operator	Administrator
Sample Name	POT ESI 155F31	Instrument	apex-III
Comment			

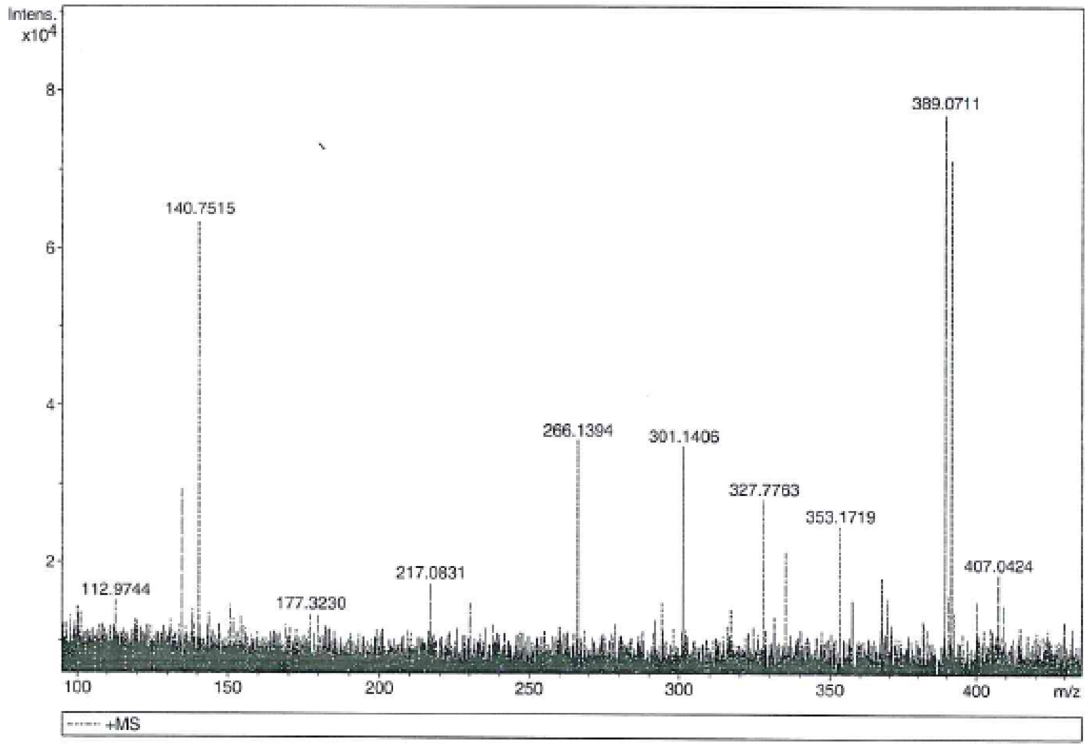

Sum Formula	Sigma	m/z	Err [ppm]	Mean Err [ppm]	Err [mDa]	rdb	N Rule	e⁻
C 18 H 23 Br 1 Na 1 O 3	0.035	389.0723	2.91	3.39	1.32	6.50	ok	even

Supplementary Figure S4: HRMS analysis of ISSF31.

HRMS analysis of ISSF31 run on a Waters Xevo G2 Q-ToF HRMS equipped with an ESI source (capillary voltage 3.0 kV, sampling cone 35 au, extraction cone 4 au, source temperature 120 °C and desolvation gas 450 °C with a flow of 650 L/h. MS mode is between 100 and 1500 Da. Experimental data reported in concordance with reported structure with reported ppm error <10.

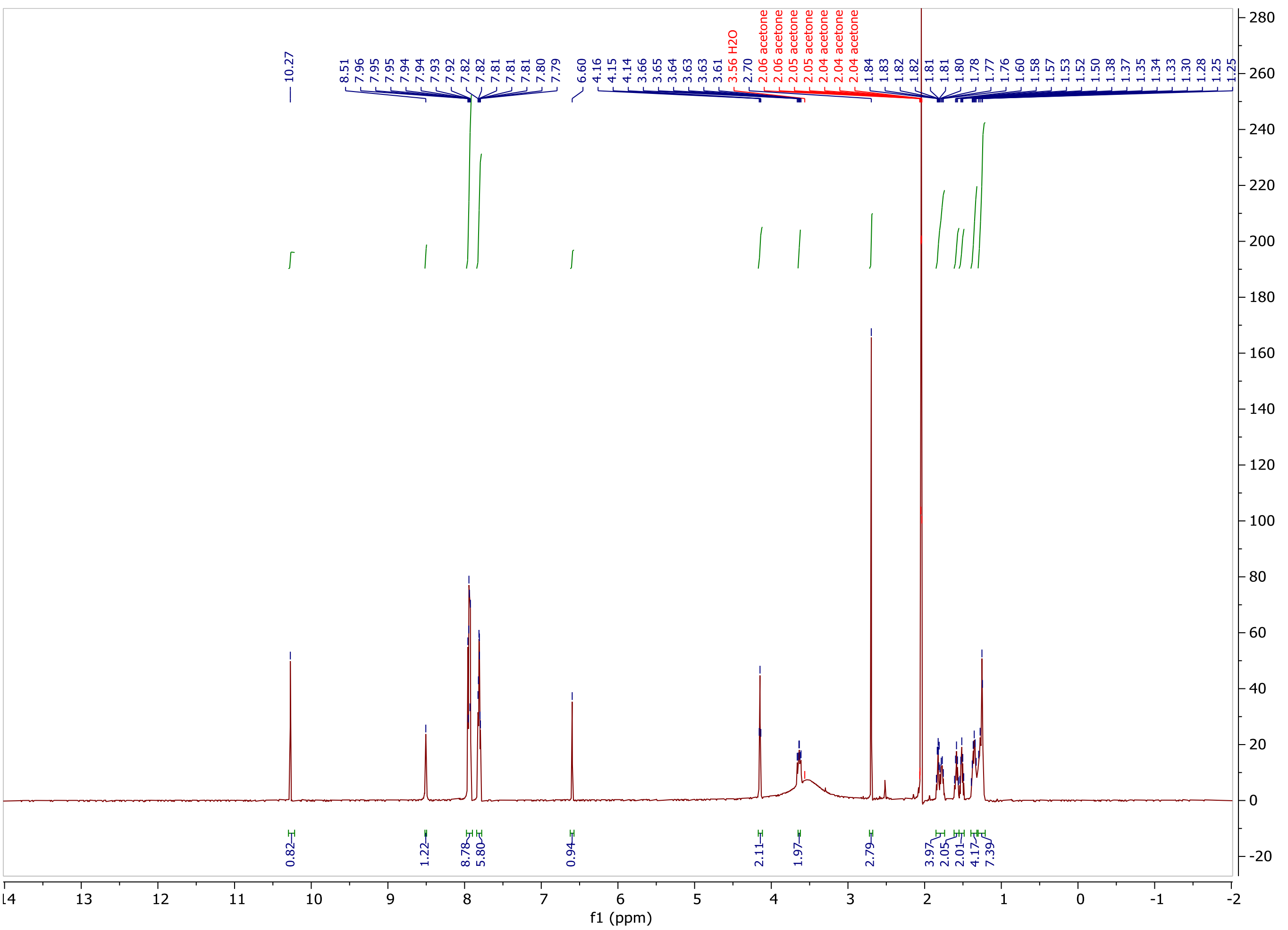

Supplementary Figure S5: ^{1}H NMR spectra of ALTOX094.

^{1}H NMR spectra of final published compound ALTOX094 in acetone-d_6 recorded on a Varian 600 MHz spectrometer. Data reported in ppm found to be in concordance with reported structure.

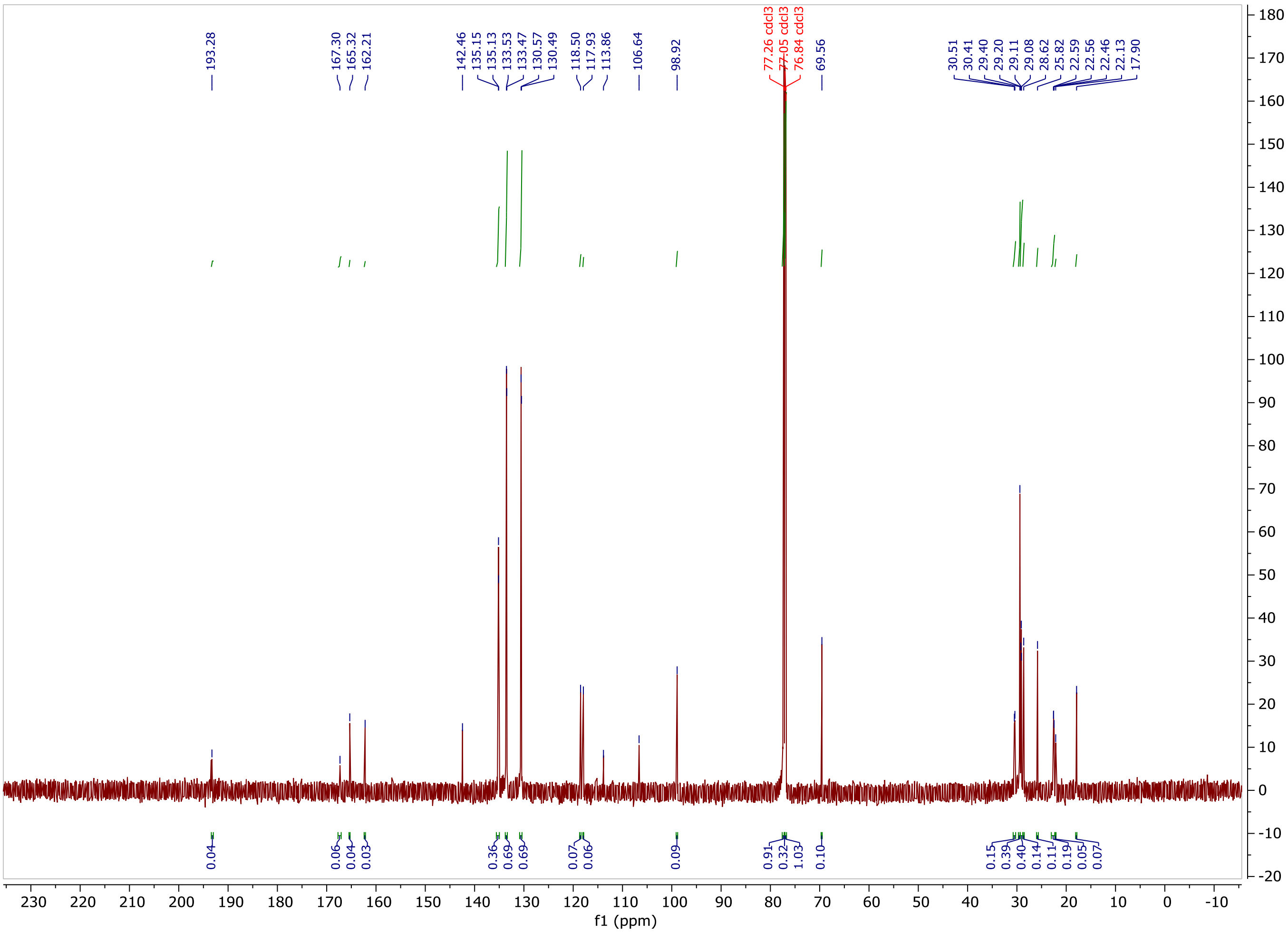

Supplementary Figure S6: ^{13}C NMR spectra of ALTOX094.

^{13}C NMR spectra of final published compound ALTOX094 in chloroform-d recorded on a Varian 600 MHz spectrometer. Data reported in ppm found to be in concordance with reported structure.

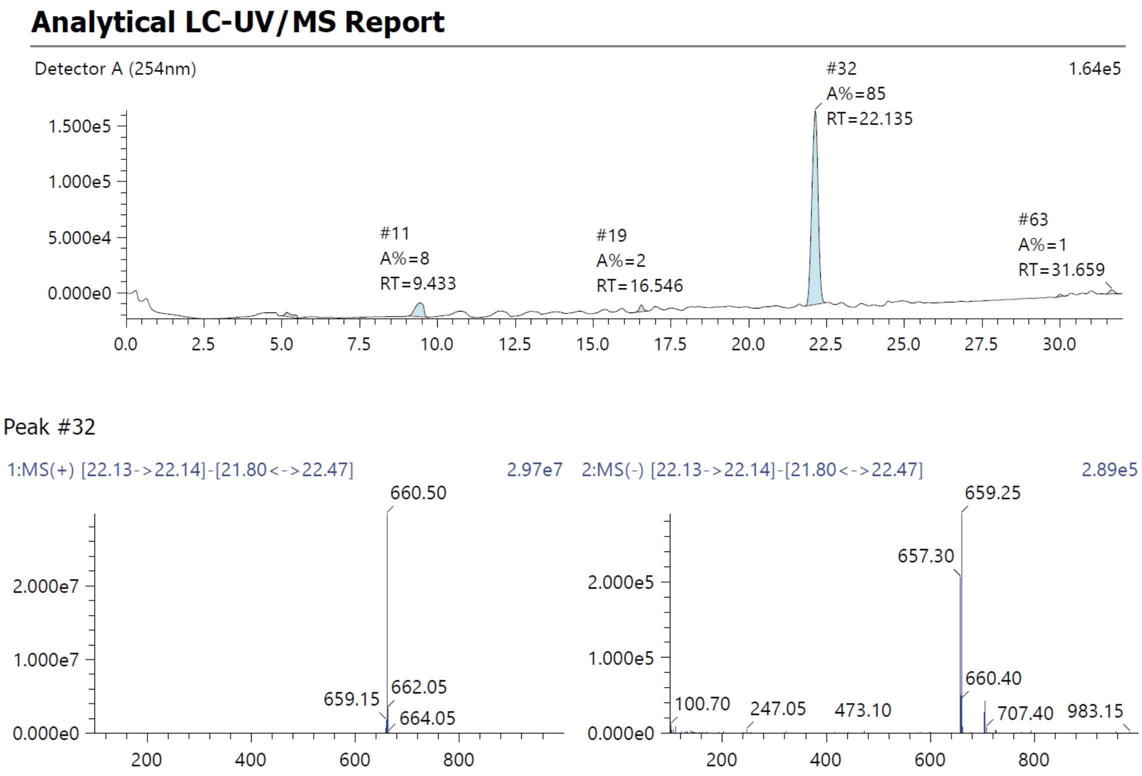

Supplementary Figure S7: LCMS analysis of ALTOX094.

LCMS analysis of reported compound ALTOX094 run on a Shimadzu LCMS-2020 system equipped with a Gemini® C18 110 Å column and a UV detector set at 254 nm. Percentage purity measurements were run using a 30 minute method in water/acetonitrile both modified with 0.1% formic acid (5 minutes 95/5, 5/95 over 20 minutes, 5 minutes 5/95). Sample concentration was very low leading to unsteady baseline and the presence of column artefacts at 9.433 min.

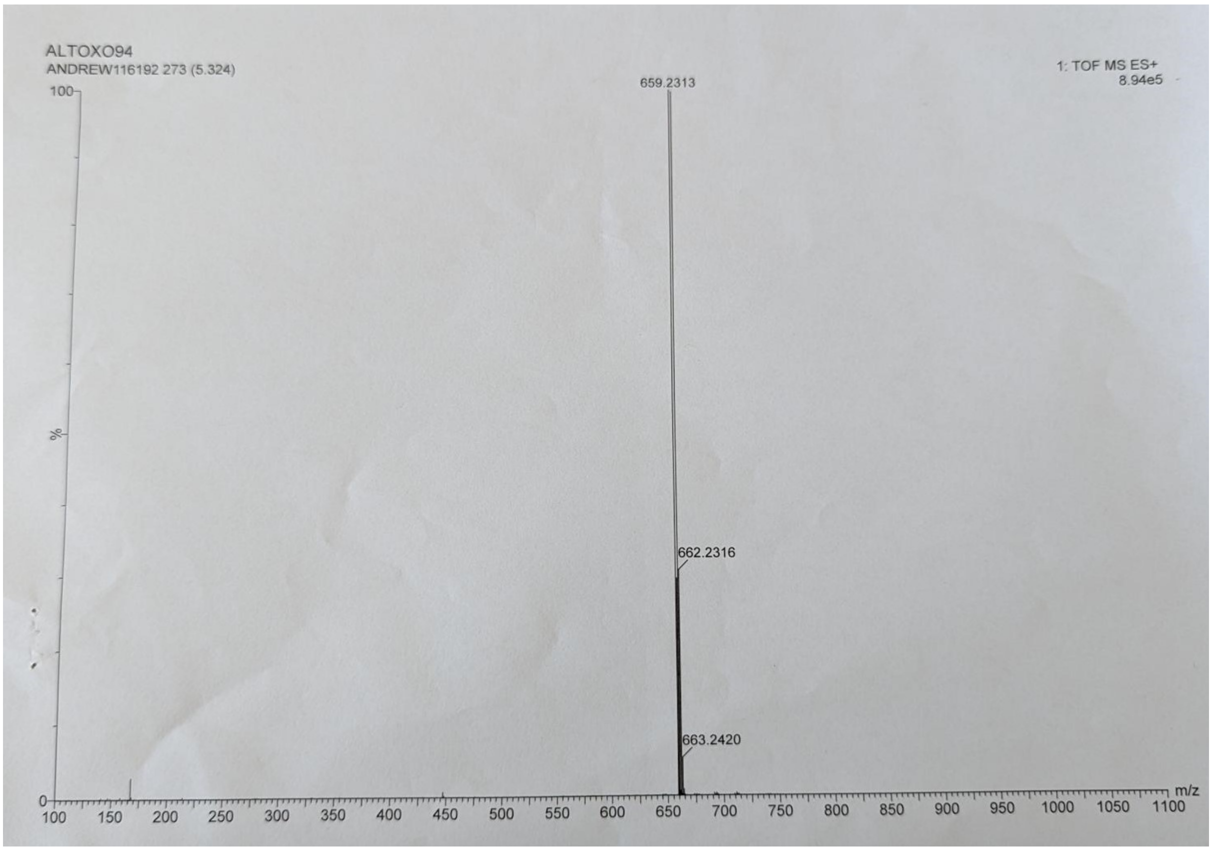

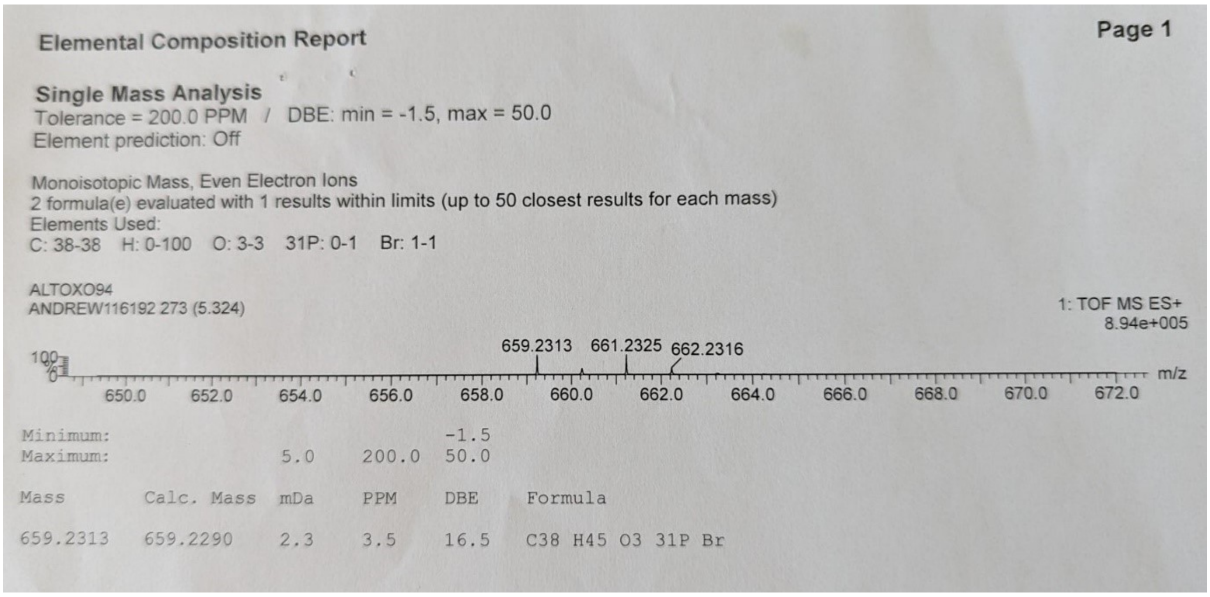

Elemental Composition Report **Page 1**

Single Mass Analysis
Tolerance = 200.0 PPM / DBE: min = -1.5, max = 50.0
Element prediction: Off

Monoisotopic Mass, Even Electron Ions
2 formula(e) evaluated with 1 results within limits (up to 50 closest results for each mass)
Elements Used:
C: 38-38 H: 0-100 O: 3-3 31P: 0-1 Br: 1-1

ALTOXO94
ANDREW116192 273 (5.324)
1: TOF MS ES+
8.94e+005

Minimum:				-1.5	
Maximum:		5.0	200.0	50.0	
Mass	Calc. Mass	mDa	PPM	DBE	Formula
659.2313	659.2290	2.3	3.5	16.5	C38 H45 O3 31P Br

Supplementary Figure S8: HRMS analysis of ALTOX094.

HRMS analysis of ALTOX094 run on a Waters Xevo G2 Q-ToF HRMS equipped with an ESI source (capillary voltage 3.0 kV, sampling cone 35 au, extraction cone 4 au, source temperature 120 ^{o}C and desolvation gas 450 ^{o}C with a flow of 650 L/h. MS mode is between 100 and 1500 Da. Experimental data reported in concordance with reported structure with reported ppm error <10.

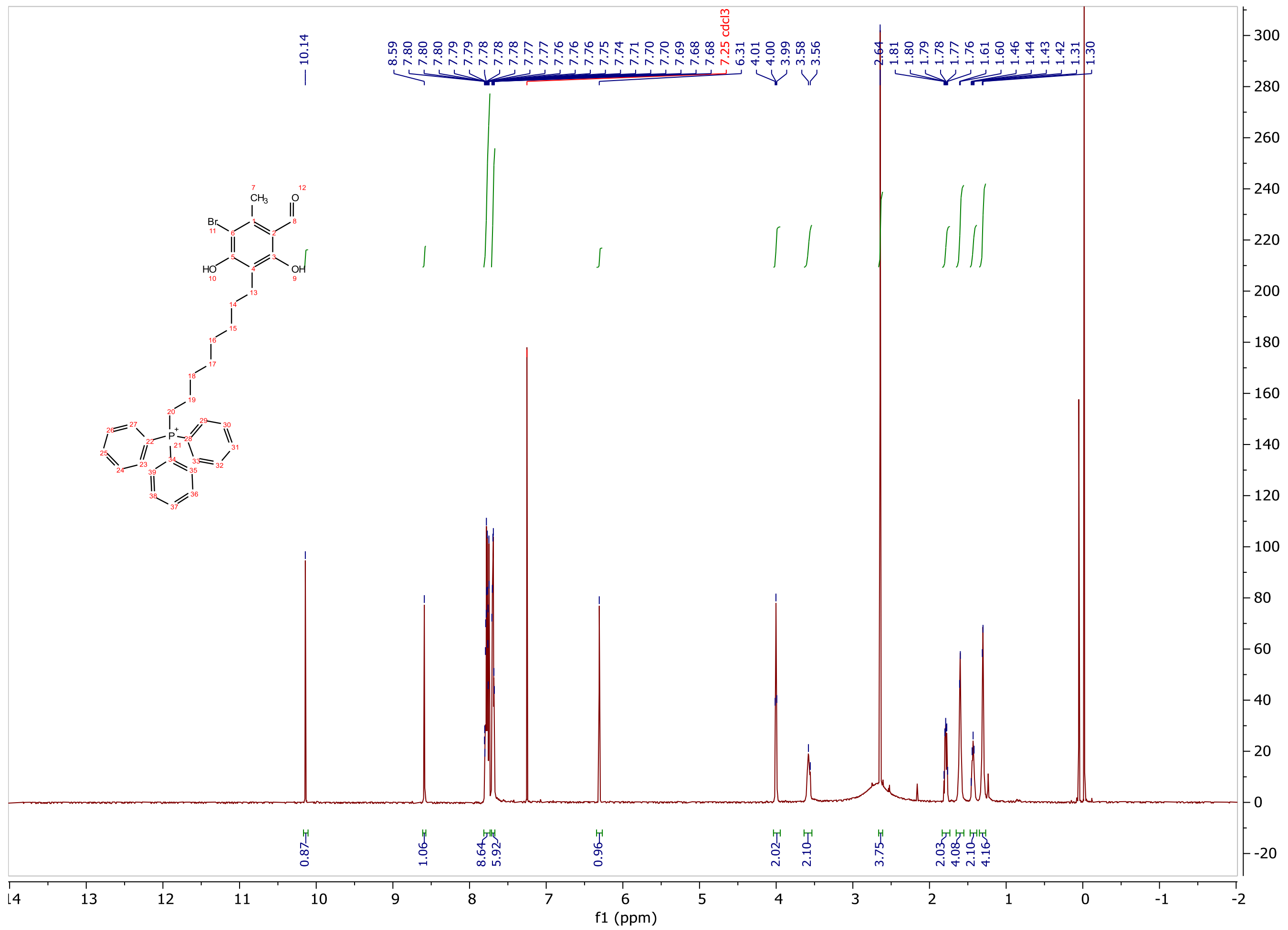

Supplementary Figure S9: ^{1}H NMR spectra of ALTOX102.

^{1}H NMR spectra of final published compound ALTOX102 in chloroform-d recorded on a Varian 600 MHz spectrometer. Data reported in ppm referenced to TMS found to be in concordance with reported structure.

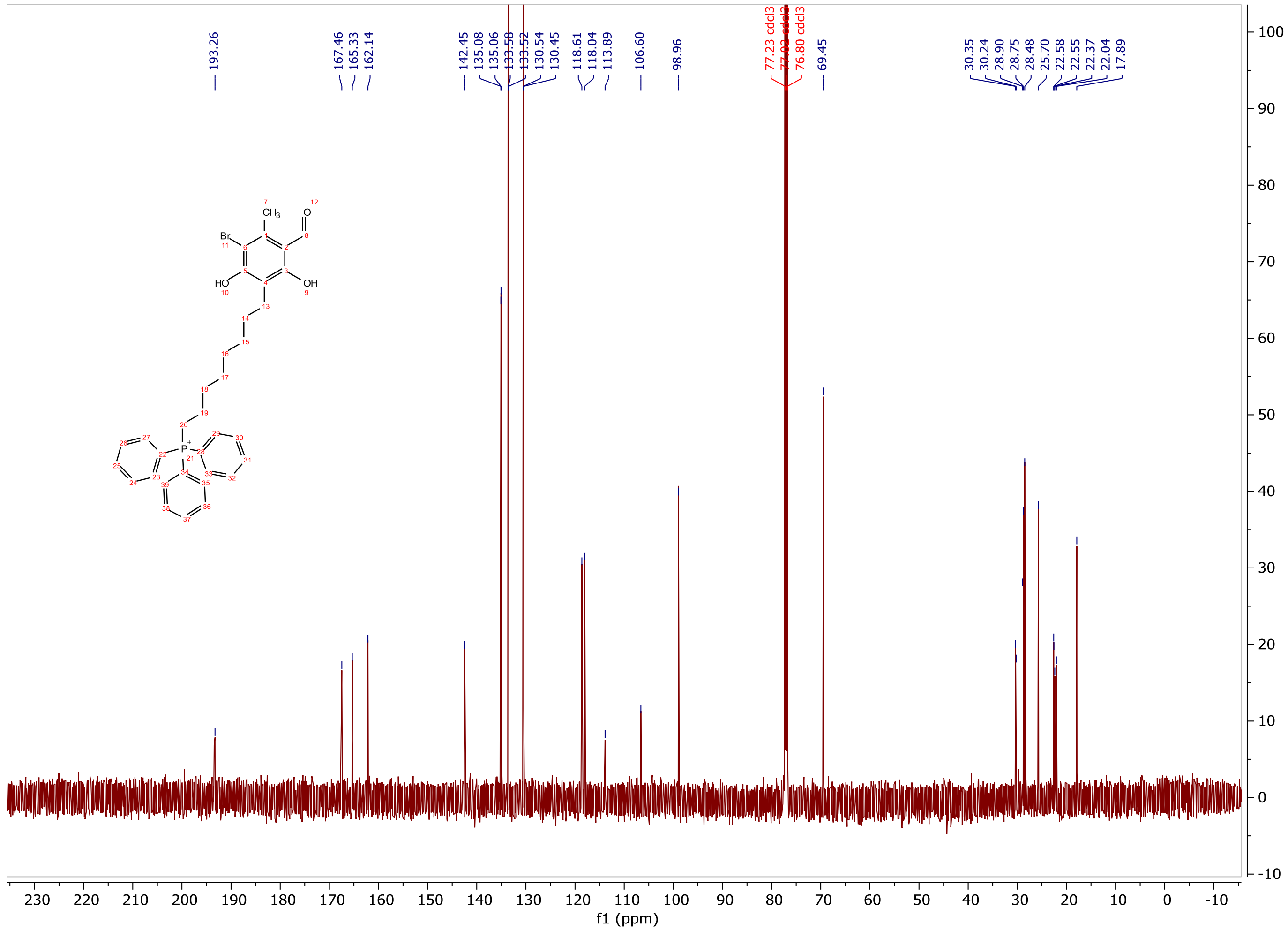

Supplementary Figure S10: ^{13}C NMR spectra of ALTOX102.

^{13}C NMR spectra of final published compound ALTOX102 in chloroform-d recorded on a Varian 600 MHz spectrometer. Data reported in ppm found to be in concordance with reported structure.

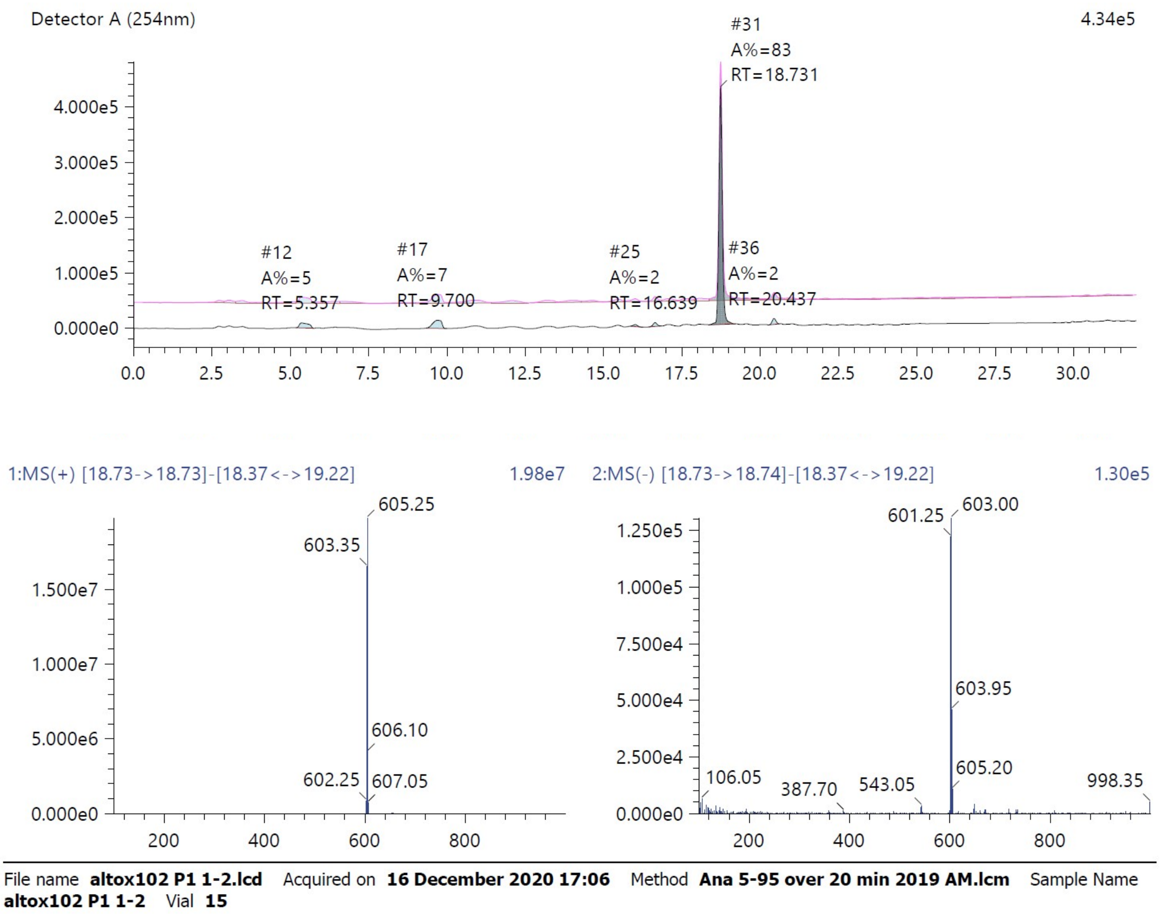

Supplementary Figure S11: LCMS analysis of ALTOX102.

LCMS analysis of reported compound ALTOX102 run on a Shimadzu LCMS-2020 system equipped with a Gemini® C18 110 Å column and a UV detector set at 254 nm. Percentage purity measurements were run using a 30 minute method in water/acetonitrile both modified with 0.1% formic acid (5 minutes 95/5, 5/95 over 20 minutes, 5 minutes 5/95). Sample concentration was very low leading to unsteady baseline and the presence of column artefacts at 9.700 min.

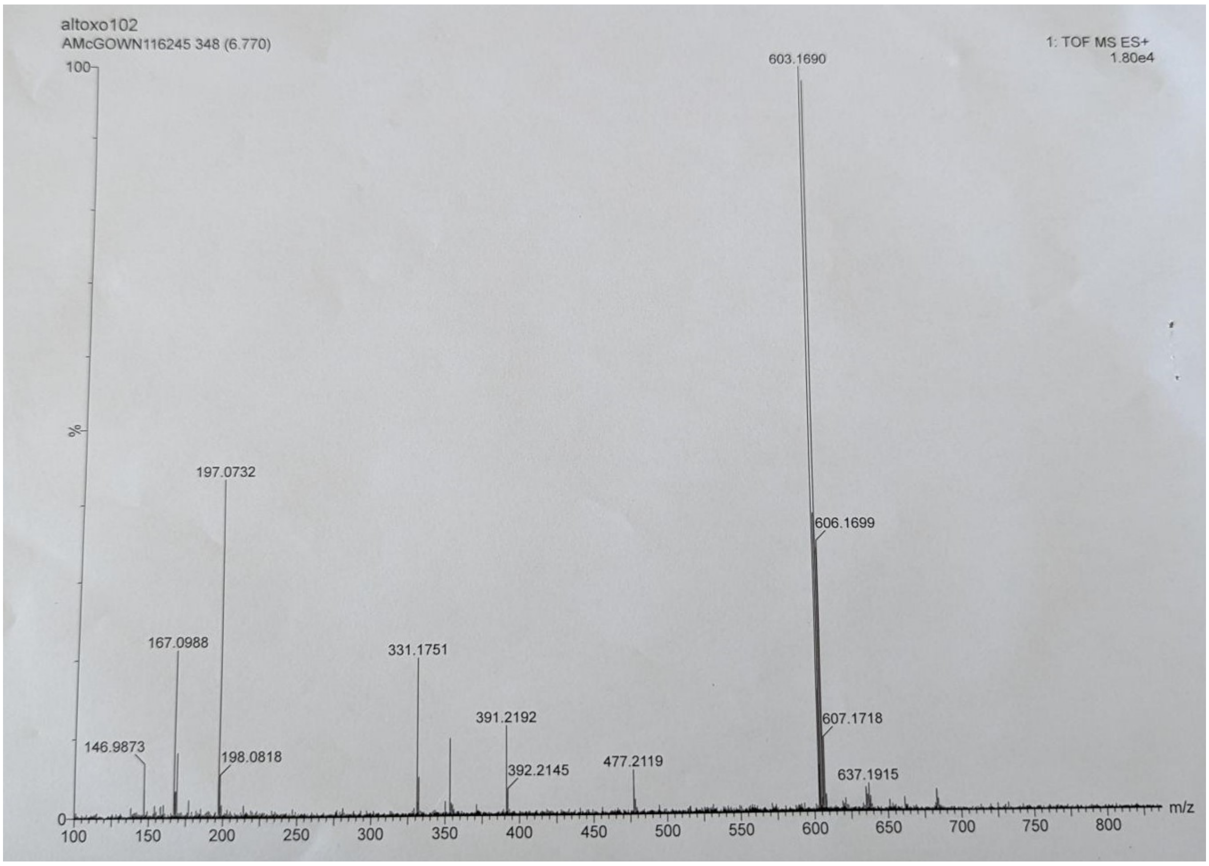

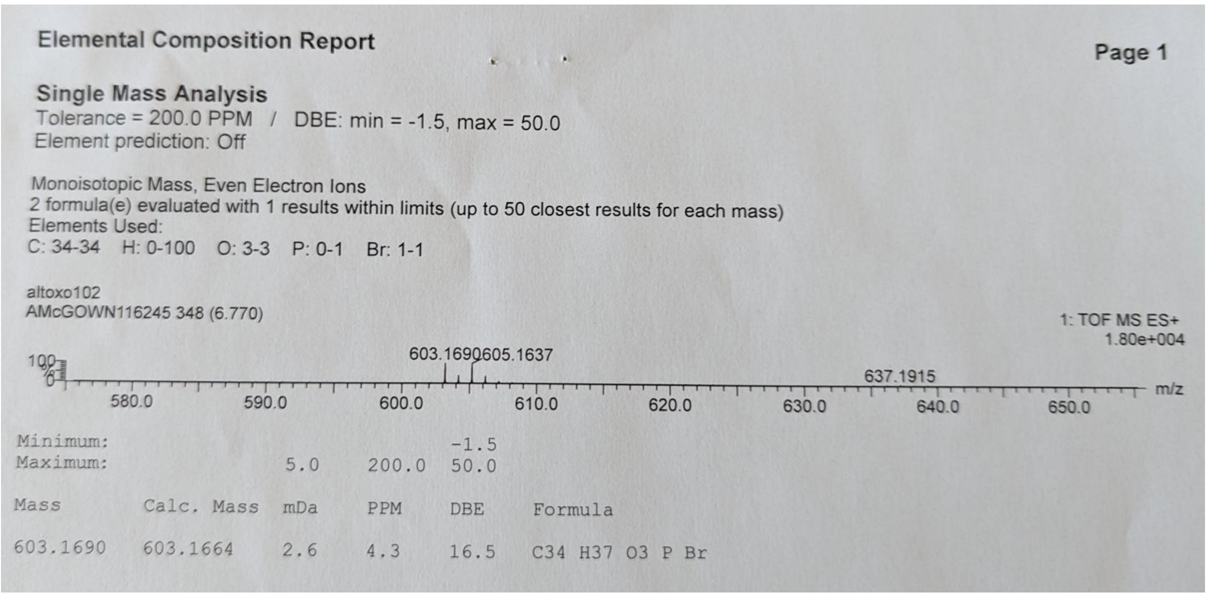

Elemental Composition Report

Page 1

Single Mass Analysis
Tolerance = 200.0 PPM / DBE: min = -1.5, max = 50.0
Element prediction: Off

Monoisotopic Mass, Even Electron Ions
2 formula(e) evaluated with 1 results within limits (up to 50 closest results for each mass)
Elements Used:
C: 34-34 H: 0-100 O: 3-3 P: 0-1 Br: 1-1

altoxo102
AMcGOWN116245 348 (6.770)
1: TOF MS ES+
1.80e+004

603.1690 605.1637 637.1915

Mass	Calc. Mass	mDa	PPM	DBE	Formula
Minimum:				-1.5	
Maximum:		5.0	200.0	50.0	
603.1690	603.1664	2.6	4.3	16.5	C34 H37 O3 P Br

Supplementary Figure S12: HRMS analysis of ALTOX102.

HRMS analysis of ALTOX102 run on a Waters Xevo G2 Q-ToF HRMS equipped with an ESI source (capillary voltage 3.0 kV, sampling cone 35 au, extraction cone 4 au, source temperature 120 ^{o}C and desolvation gas 450 ^{o}C with a flow of 650 L/h. MS mode is between 100 and 1500 Da. Experimental data reported in concordance with reported structure with reported ppm error <10.

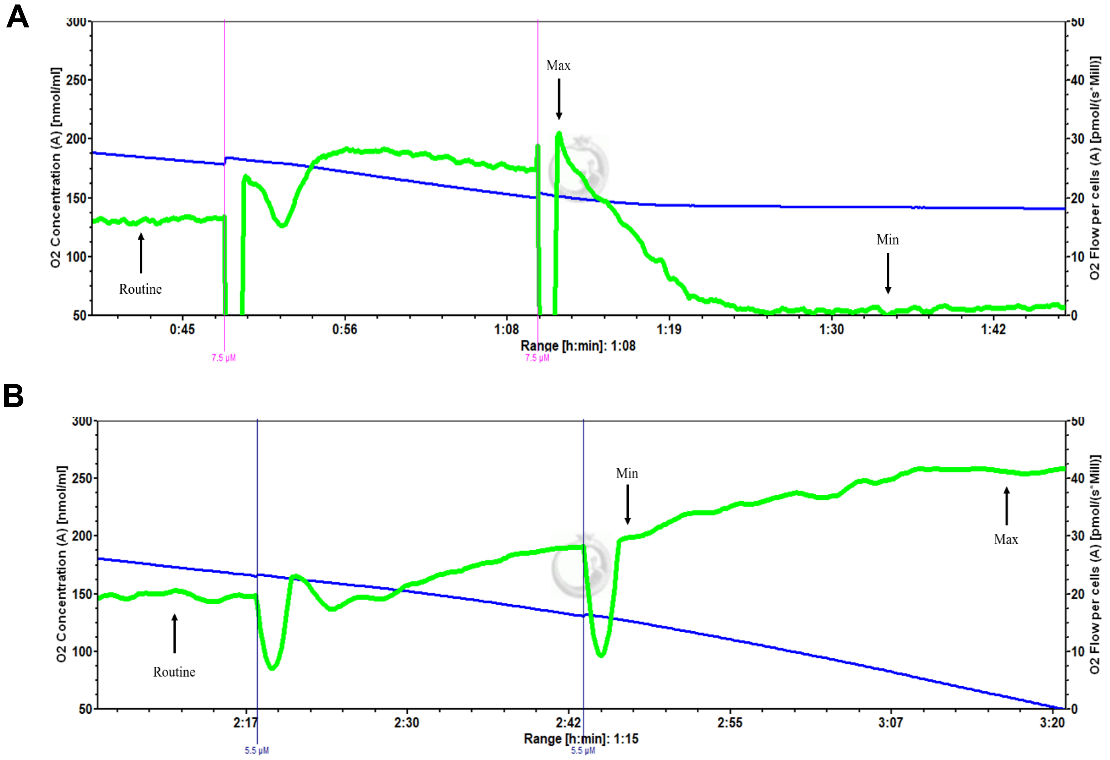

Supplementary Figure S13: Respiratory profiles of C. neoformans exposed to ALTOX drugs.
Representative example of respiration in H99 and *Δaox1* determined using HRR. Chambers were inoculated with 1 x 10^6 cells after 24 h growth and treated with either ALTOX094 or ALTOX102 where indicated to a final concentration of the MIC90 for both drugs. (**A**) *Δaox1* + ALTOX094**, (B)** *Δaox1* + ALTOX102.

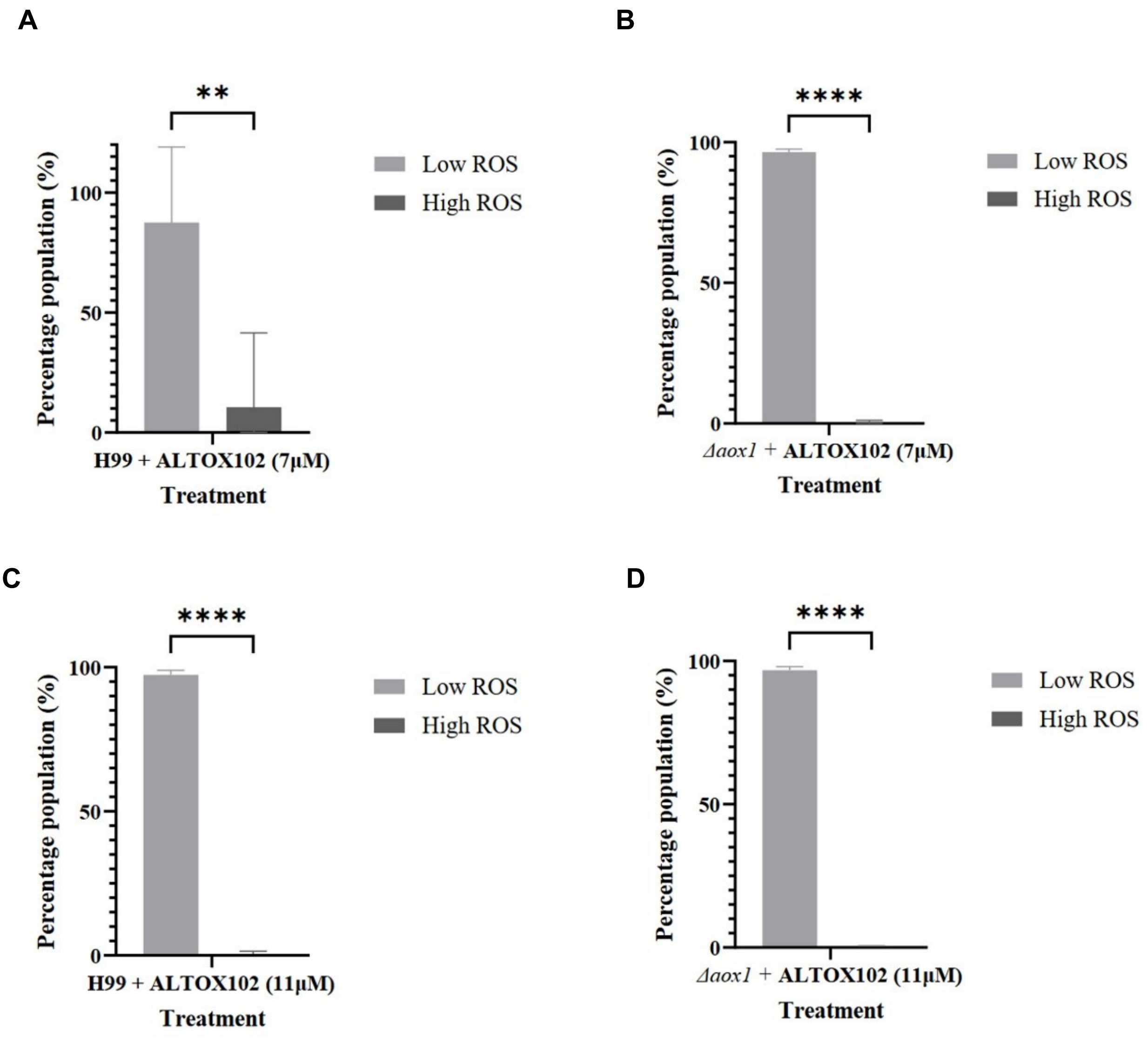

Supplementary figure S14: Flow cytometry assay of C. neoformans exposed to ALTOX094 and ALTOX102 treatment at the MIC90.
C. neoformans strains were stained with PI and analysed via Flow Cytometry after a 2 h incubation with ALTOX094 or ALTOX102 at the given MIC90. Percentage necrosis was measured via fluorescence in V1-R for (**A**) H99 + 7µM ALTOX102 (**B**) *Δaox1* + 7µM ALTOX102 (**C**) H99 + 11µM ALTOX102 (**D**) *Δaox1* + 11µM ALTOX102. Significance was calculated using Dunnett's multiple comparisons test following a one-way ANOVA in GraphPad Prism. ** <0.005, *** <0.0005, **** <0.0001, where P = 0.05. Error bars represent ± SD. n = 9

A

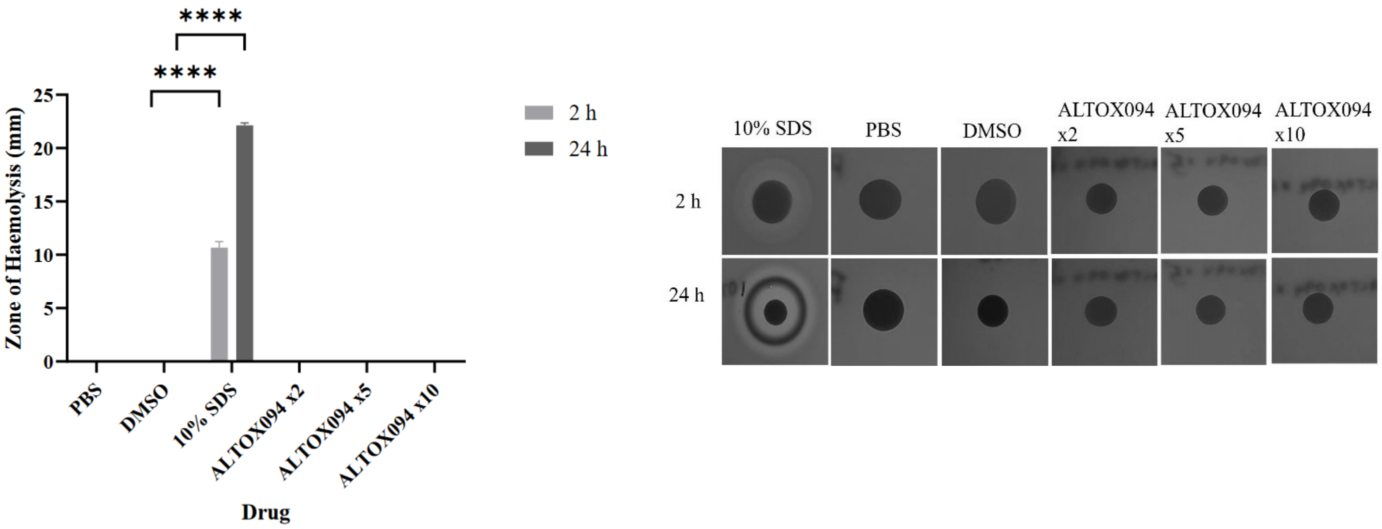

B

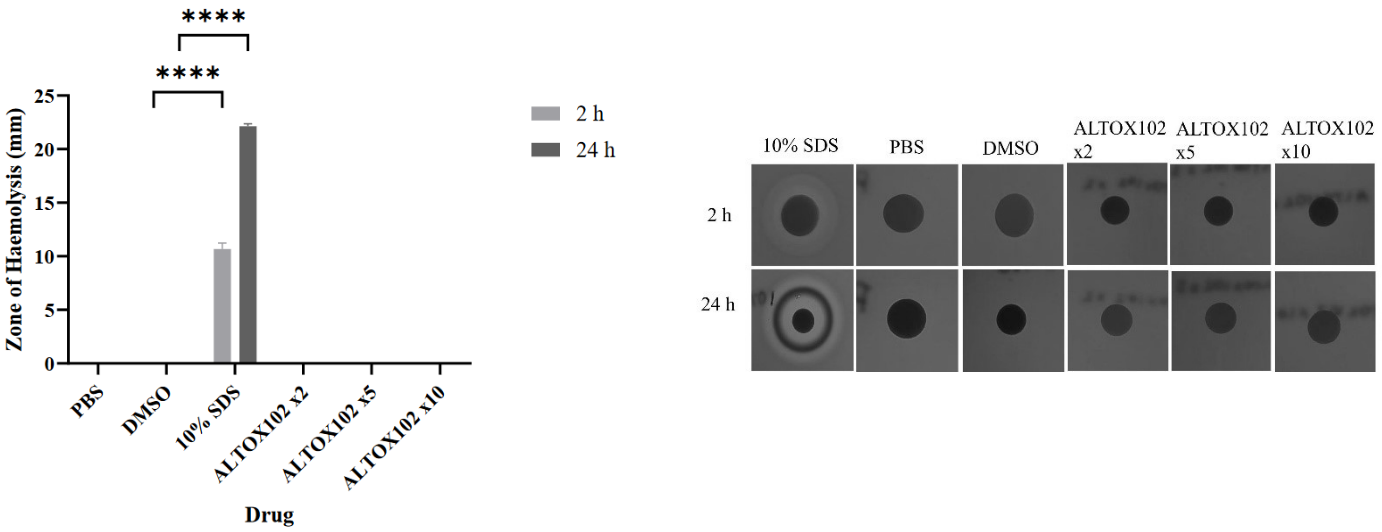

Supplemental Figure S15: Haemolytic effect of ALTOX drugs. ALTOX094 and ALTOX102 were administered to filter paper disks on agar plates containing 5% sheep blood at the MIC90 and incubated at 37°C. The zone of haemolysis (ZOH) was measured after 2 h exposure and 24 h exposure. (**B**) ALTOX094 and (**C**) ALTOX102 was administered at concentrations x2, x5 and x10 the MIC90. The zone of haemolysis (ZOH) was measured after 2 h exposure and 24 h exposure. Significance was calculated using Dunnett's multiple comparisons test following a two-way ANOVA in GraphPad Prism. **** < 0.0001 where P = 0.05. n = 3

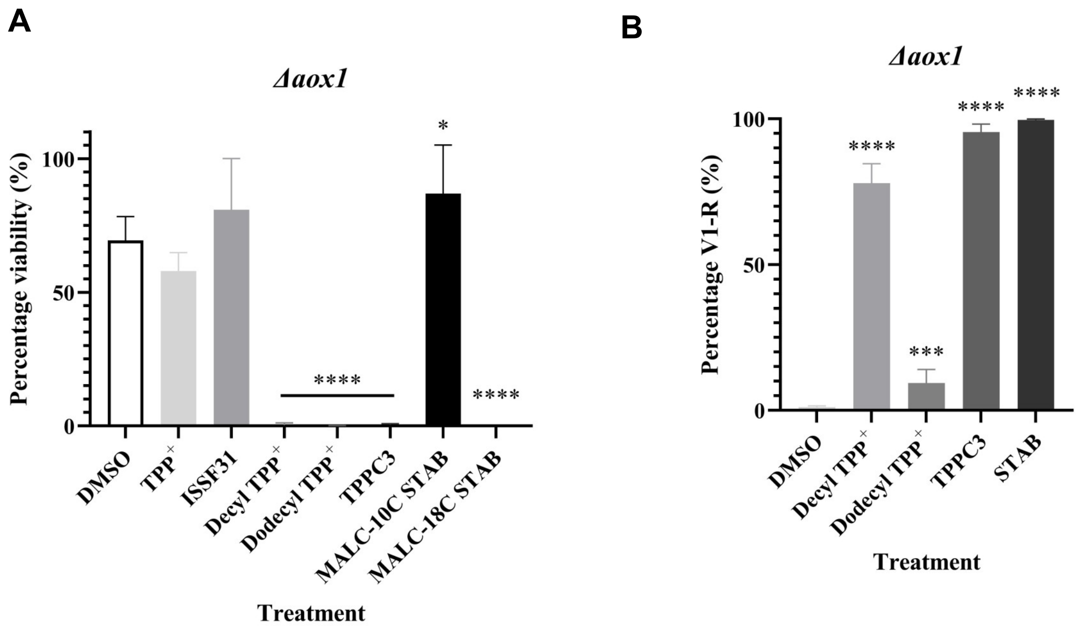

Supplemental Figure 16: Screening of ALTOX094 reference compounds and their effect on C. neoformans growth, viability and respiration.

(**A**) The viability of Aox1 null mutant (*Δaox1*) *C. neoformans* cells exposed to TPP^+ and ISSF331, and ALTOX094 reference compounds Decyl TPP^+, Dodecyl TPP^+, TPPC3, and MALC reference compounds MALC-10C STAB and MALC-18C STAB. Cultures were grown in YPD containing each compound at the MIC90 for 48 h at 37°C. (**B**) Aox1 null mutant (*Δaox1*) *C. neoformans* was stained with PI and analysed via Flow Cytometry after a 2 h incubation with ALTOX094 reference compounds. Percentage necrosis was measured via fluorescence in V1-R in comparison to a DMSO control. Significance was calculated using Dunnett's multiple comparisons test following a one-way ANOVA in GraphPad Prism. * <0.05, **<0.005, *** <0.0005, **** <0.0001, where P = 0.05. Error bars represent ± SEM. n = 9

Drug	Structure	Effect on *C. neoformans*	Mechanism of Action	MIC90	Ref	Notes
**Base molecule: Colletochlorin B**	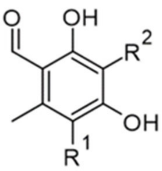					R1/R2 can be substituted with Br, Cl or other given alkyl groups
**ALTOX094**	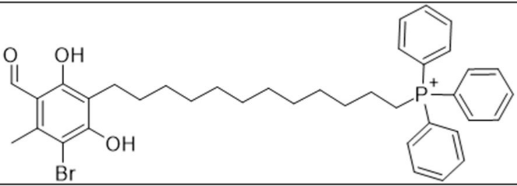	Loss of growth, respiration and viability	Necrotic action against *C. neoformans*	15µM	Fig. 4,5 S2, S3, S5	Non-haemolytic and does not affect *G. mellonella* mortality (Fig. 6.1)
**ALTOX094 Reference Drug**						
Triphenylphosphonium (TPP⁺)	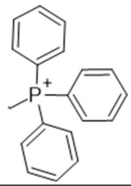	No negative effect on growth, respiration or viability	No action against *C. neoformans*	15µM	Fig. 8, S7, S8, S9	Base compound for drug targeting. Non-haemolytic
TPPC3	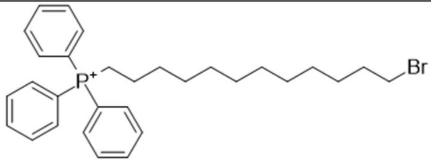 (12-bromododecyl)triphenylphosphonium	Loss of growth, respiration and viability	Necrotic action against *C. neoformans*	15µM	Fig. 8, 9, S7, S8, S9,	Non-haemolytic
Decyl TPP⁺	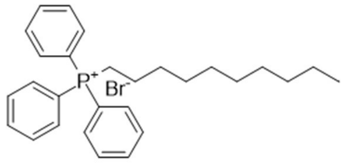 (1-Decyl)Triphenylphosphonium bromide	Loss of growth, respiration and viability	Necrotic action against *C. neoformans* but mixed populations of ROS (high and low)	15µM	Fig. 8, 9, S7, S8, S10	Non-haemolytic
Dodecyl TPP⁺	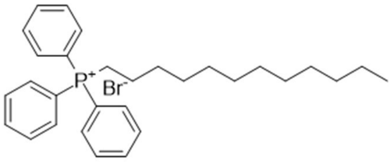 (1-Dodecyl)triphenylphosphonium bromide	Loss of viability but no inhibition of respiration	Non-necrotic cell death with mixed populations of ROS	15µM	Fig. 8, 9, S7, S8, S10	Non-haemolytic. Action may be dose-dependent but independent of respiratory inhibition

Table S1—Summary of structural function analysis of ALTOX094 and its reference compounds

**Drug**	**Structure**	**Effect on *C. neoformans***	**Mechanism of Action**	**MIC90**	**Ref**	**Notes**
**ALTOX102**	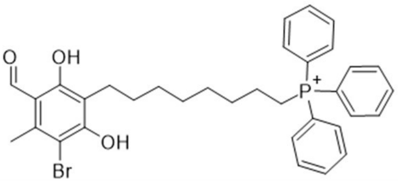	Dose-dependent effect on growth, viability and respiration. High MIC90 induces loss of viability but increase in respiration	Non-necrotic cell death against *C. neoformans* with low ROS levels	7 -11μM	Fig. 4,5 S2, S3	Non-haemolytic but does affect *G. mellonella* mortality (Fig. 6.1). Action may be dose-dependent
**ALTOX102 Reference Drug**						
Octyl TPP⁺	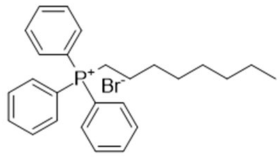 (1-Octyl)triphenylphosphonium bromide	No negative effect on growth, viability or respiration. Dose-dependent respiratory increase and mild fungistatic action observed in wildtype cells at high MIC90	Mild fungistatic action against *C. neoformans*	7 -11μM	Fig. 8, S7, S8, S11	Non-haemolytic
**MALC reference drug**						
MALC – 18C STAB	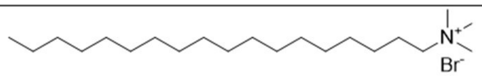 Trimethylstearylammonium Bromide MALC	Loss of growth and viability but respiration increased	Necrotic action against *C. neoformans*	15μM	Fig. 8, 9, S7, S8, S12	Non-haemolytic. Necrotic activity likely to involve membrane targeting
MALC – 10C STAB	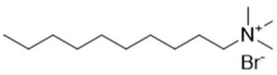	No negative effect on respiration, growth or viability	No action against *C. neoformans*	15μM	Fig. 8, 9, S7, S8, S12	Non-haemolytic

Table S2—Summary of structural function analysis of ALTOX102 and its reference compounds
